# Human cytomegalovirus strain-specific differences in protein expression of type I IFN pathway proteins do not impact virus replication

**DOI:** 10.1099/acmi.0.001104.v3

**Published:** 2026-02-19

**Authors:** Katie A. Latham, Timothy K. Soh, Richard J. Stanton, Jens B. Bosse, Steve Goodbourn, Blair L. Strang

**Affiliations:** 1 Institute of Infection & Immunity, St George’s School of Health and Medical Sciences, City St George’s University of London, London, UK; 2 Centre for Structural Systems Biology, Hamburg, Germany; 3 Hannover Medical School, Institute of Virology, Hannover, Germany; 4 Cluster of Excellence RESIST (EXC 2155), Hannover Medical School, Hannover, Germany; 5 Leibniz Institute of Virology (LIV), Hamburg, Germany; 6 Division of Infection and Immunity, Cardiff University School of Medicine, Cardiff, UK

**Keywords:** alphafold, cytomegalovirus, human, IFN

## Abstract

The type I IFN response is crucial for cells to restrict viral replication during infection. Many viruses, including human cytomegalovirus (HCMV), have evolved mechanisms to antagonize the type I IFN response. We have previously observed an increase in protein expression of certain IFN-stimulated genes when comparing the high-passage HCMV strain AD169 to the low-passage strain HCMV Merlin, suggesting that AD169 is defective in its ability to inhibit type I IFN function. To better understand HCMV interaction with the type I IFN response, we examined expression of cellular and viral proteins expressed in Merlin- and AD169-infected cells associated with IFN production and signalling. HCMV IFN antagonists expressed by both viruses had differences in amino acids throughout their protein sequences, although analysis using AlphaFold revealed that there was likely to be no obvious differences in the overall structure of these proteins. Analysis of quantitative mass spectrometry datasets showed modest differences in the expression of cellular IFN-associated proteins between strains. Contrary to previously reported data, we found no obvious loss of IRF3 expression, though this may be due to experimental differences between studies. These data revealed that multiplicity of infection was an important factor in IRF3 expression. We found little or no statistical difference in the production of IFN-*β* RNA between Merlin- and AD169-infected cells in reverse transcriptase quantitative PCR assays and little or no statistical difference in replication of AD169 and Merlin in virus replication assays. Overall, these data suggest that different strains of HCMV have different, albeit modest, abilities to influence the expression of type I IFN pathway proteins during infection. However, this had no overall impact on the ability of different strains to produce a type I IFN or to replicate.

## Data Summary

All data associated with this work are reported within the article and supplementary file.

## Introduction

Human cytomegalovirus (HCMV), also known as human herpesvirus 5, is a member of the *Betaherpesvirinae* family [1,2]. HCMV is a widespread human pathogen with a global seroprevalence of 60–90%, varying depending on socioeconomic background [3]. Infection of immunocompetent individuals is typically asymptomatic, and infection of immunocompromised individuals (such as those with cancer, AIDS or organ transplant recipients) can lead to notable morbidity or mortality [4]. Additionally, periodic reactivation of latent HCMV due to ageing or immunosuppression can also lead to morbidity or mortality, which contributes to a life-long socio-economic burden [5,6]. HCMV is the most common infectious cause of congenital disease affecting ~1 in 200 pregnancies [4]. Transmission of HCMV can occur from mother to foetus, who has an immature immune system, and can lead to severe disease [7]. Infection during pregnancy, or in neonates, can cause birth defects (such as small weight, jaundice and microcephaly) or can lead to developmental disabilities such as hearing loss [8,9].

 In order for HCMV to replicate, it must overcome human host defences, including the innate immune response to infection [10]. The innate immune response is an essential non-specific mechanism that acts as the first line of defence against pathogens and aims to inhibit viral replication [10,11]. One important part of the innate immune response is the IFN pathways. There are three classes of IFN proteins (types I, II and III), each with specific functions [12,13]. There are two distinct, but interconnected, type I IFN pathways: IFN production and IFN signalling. The IFN production pathway is initiated by recognition of the virus by pattern recognition receptors, which triggers distinct intracellular signalling pathways [12]. These pathways result in activation of the transcription factors: nuclear factor-κB (NF-κB) and IFN regulatory factor 3 (IRF3) [14]. These activated transcription factors localize to the nucleus where they bind to the promoter region of the type I IFN genes and elicit the transcription and expression of IFN-*α* and IFN-*β* [13]. IFN-*α* and IFN-*β* are secreted from the cell and bind to their cognate receptor (IFNAR) in an autocrine or paracrine manner [15]. This binding activates IFN signalling via the Janus protein tyrosine kinase (JAK)/signal transducers and activators of transcription (STAT) proteins. These proteins activate transcription of at least 400 type I IFN-stimulated genes (ISGs) that possess antiviral functions [13,16].

HCMV is a highly successful pathogen, partly due to its ability to antagonize the type I IFN response. HCMV encodes at least 170 proteins [17,19]. Of those proteins that have been characterized, there are currently seven that have been documented to be involved in IFN antagonism: US9, UL26, UL31, UL82 (pp71), UL83 (pp65), UL122 (IE2) and UL123 (IE1). These proteins target type I IFN production or signalling (reviewed in 5,18)[20].

However, it is possible that our understanding of the interaction between HCMV and the type I IFN response is incomplete. This may be due to the study of different HCMV strains and cell types in different studies. Continual passaging of several HCMV strains has led to mutation across their genomes and the loss of genomic regions. For example, the well-known and highly passaged HCMV strain AD169 contains many nucleotide changes compared to wild-type HCMV strains and lacks a 20 kb region that has been deleted from its genome [21]. Presently, many investigations are underway to understand the genetic complexity of HCMV. Low-passage strains, such as Merlin, are thought to be more representative of HCMV strains found in clinical settings. However, to our knowledge, there has been no direct comparison of high and low passage HCMV strains and their interaction with the type I IFN system. Additionally, to our knowledge, there had been no direct comparison of HCMV and its interaction with the type I IFN system in adult and foetal cells.

Differences in global protein expression between strains such as AD169 and the low-passage wild-type strain Merlin have been observed in several large quantitative mass spectrometry studies of the HCMV proteome [22,23], although comparison of Merlin and AD169 interaction with the IFN system has not been investigated. Furthermore, a report from one of our laboratories demonstrated a difference in expression of at least two ISGs (MxA and ZAP-S) in AD169-infected cells compared to Merlin-infected cells, suggesting differences in ISG expression between these two strains [23]. Therefore, we set out to investigate strain-specific differences in AD169- and Merlin-infected cells, analysing antagonists of type I IFN expressed by both strains, the expression of cellular proteins associated with the type I interferon response and the production of a type I IFN in infected cells in both adult and foetal fibroblasts.

## Methods

### Cells and viruses

Human foreskin fibroblast (HFF) cells were obtained from the American Type Culture Collection no. CRL-1684 (ATCC, Manassas, VA). Human foetal foreskin fibroblast (HFFF-TERT) cells were gifted from Richard Stanton at Cardiff University [24]. All cell lines were cultured in Dulbecco’s Modified Eagle Medium (DMEM) (Sigma) containing 10% FBS (Sigma) at 37 °C and 5% CO_2_. HCMV strain Merlin(R1111), which contains deletions in HCMV genes encoding RL13 and UL128 to allow release of cell-free virus, was used as the low-passage wild-type-like strain. HCMV strain AD169 was a gift from Donald Coen (Harvard Medical School). Sendai virus (SeV) strain Cantrell in amnioallantoic fluid (Charles River Avian products) was used as a positive control for type I IFN induction.

### Determination of HCMV titre

HCMV titres were determined by serial dilution of HCMV supernatant onto HFF monolayers, which were then overlayed with DMEM containing 5% FBS and 0.6% methylcellulose. After 14 days of incubation at 37 °C and 5% CO_2_, cells were fixed with 100% methanol and stained with 1% crystal violet. Plaques in the infected monolayers were counted, and HCMV titre was expressed as p.f.u. ml^−1^.

### Sequence analysis of HCMV proteins

Protein sequences from each HCMV strain analysed were obtained from the National Center for Biotechnology Information (NCBI). HCMV strains included were Merlin(R1111) (NC_006273.2), AD169 (BK000394.5), TB40/E (EF999921.1), JP (GQ221975.1), JHC (HQ380895.1), HAN1 (JX512199.1), Toledo (KY002201.1), Davis (JX512198.1), Towne (FJ616285.1) and TR (KF021605.1). Protein sequence alignments were performed using ClustralΩ (version 1.2) [25]. TreeDyn (version 198.3) on the server Phylogeny.fr was used to construct phylogenetic trees [26].

### AlphaFold protein structure prediction

Sequence alignments were performed with MMseqs2 and HHblits. Protein structure predictions were made with AlphaFold version 2.3.0 [27]. For each protein, five models were generated and optimized by testing the consistency of the pLDDT scores associated with every model. PyMOL version 3.1.3 was used for visualization of structures.

### Infection of cells for Western blotting analysis

HFF and HFFF-TERT cells were infected with Merlin(R1111) or AD169 at a multiplicity of infection (MOI) of 1 for 1 h at 37 °C and 5% CO_2_. After infection, viral supernatant was removed, and DMEM with 10% FBS was added for the time indicated in the figures at 37 °C and 5% CO_2_: the conditions under which cells were treated and/or infected are outlined in figure legends. In each independent experiment, a single biological replicate was examined.

### Western blotting

The conditions under which cells were treated and/or infected are outlined in the figure legends. Cells were lysed using Laemmli buffer solution with 5% *β*-mercaptoethanol and incubated at 95 °C for 5 min and then stored until use at −20 °C. Proteins were separated using SDS-PAGE on 8% or 10% polyacrylamide gels and transferred to Hybond 0.45 µm polyvinylidene difluoride membrane (GE Healthcare Amersham). After blocking with 5% dry milk in tris-buffered saline with Tween 20 (TBS-T), the membranes were incubated overnight at 4 °C with primary antibodies: IRF3 (Cell Signalling, 11904 and 4302, both 1:1,000 dilution), STAT2 (Abcam, ab32367, 1:5,000 dilution) and *β*-actin (Sigma, A5441, 1:5,000 dilution). The membranes were then washed with TBS-T and incubated for 1 h at room temperature with secondary antibodies: Dylight 800 Goat Anti-Rabbit IgG (Abbkine, A23920) or Dylight 800 Goat Anti-Mouse IgG (Abbkine, A23910). Proteins were visualized using the Odyssey Li-COR imager.

### Reverse transcriptase quantitative PCR

Total RNA from uninfected at 0 h, HCMV-infected or Sendai virus-infected HFF or HFFF-TERTs was extracted using TRIzol^®^ (Invitrogen) after 6 or 24 h post-infection. Reverse transcriptase quantitative PCR (qPCR) was performed using Luna^®^ Universal One-Step RT-qPCR Kit (NEB) using primers specific for IFNB1 (F- ATTGCTCTCCTGTTGTGCTT, R-TCTCCTCAGGGATGTCAAAGT) and glyceraldehyde-3-phosphate dehydrogenase (GAPDH) (F-GAAGGTGAAGGTCGGAGTC, R-GAAGATGGTGATGGGATTTC). Amplification was performed in a 20 µl reaction mixture containing <1 µg total RNA in <7.4 µl, 1 µl Luna WarmStart^®^ RT enzyme mix and 0.8 µl of each primer at 10 µM and made up to a final volume of 20 µl with ddH_2_O. The reaction consisted of 1 cycle of 10 min at 55 °C, 1 cycle of 1 min at 95 °C and 35 cycles of 10 s at 95 °C and 1 min at 60 °C. Amplification values were extracted, and the IFNB1 value was normalized to its respective GAPDH value for each different RNA sample analysed. In each independent experiment, a single biological replicate of each RNA sample was examined. Statistical analyses were performed using GraphPad Prism software (version 10.2.3). Two-tailed unpaired t-tests were used to evaluate statistical significance.

### Virus replication assay

HFF and HFFF-TERT cells were infected with Merlin(R1111) or AD169 at an MOI of 1 for 1 h at 37 °C and 5% CO_2_, after incubation viral supernatant was removed and DMEM with 10% FBS was added for 144 h at 37 °C and 5% CO_2_. After 144 h, supernatants were collected and stored in LN_2_. Viral supernatants were analysed using a plaque assay to determine viral titre, as outlined above. In each independent experiment, a single biological replicate was examined. The limit of detection in these assays was the ability to visualize a single plaque in the lowest dilution of supernatant onto cells (a final titre of 1×10^2^ p.f.u. ml^−1^). Statistical analyses were performed using GraphPad Prism software (version 10.2.3). Two-tailed unpaired t-tests were used to evaluate statistical significance.

## Results

### Differences in expression of viral and cellular proteins involved in IFN production, IFN signalling and IFN antagonism

 Comparisons of proteomes during infection with different HCMV strains have shown differences in cellular protein expression [22,23, 28]. We set out to understand if differences in the protein expression of HCMV type I IFN antagonists accounted for previously documented differences in expression of proteins involved in type I IFN production and signalling.

**Fig. 1.**
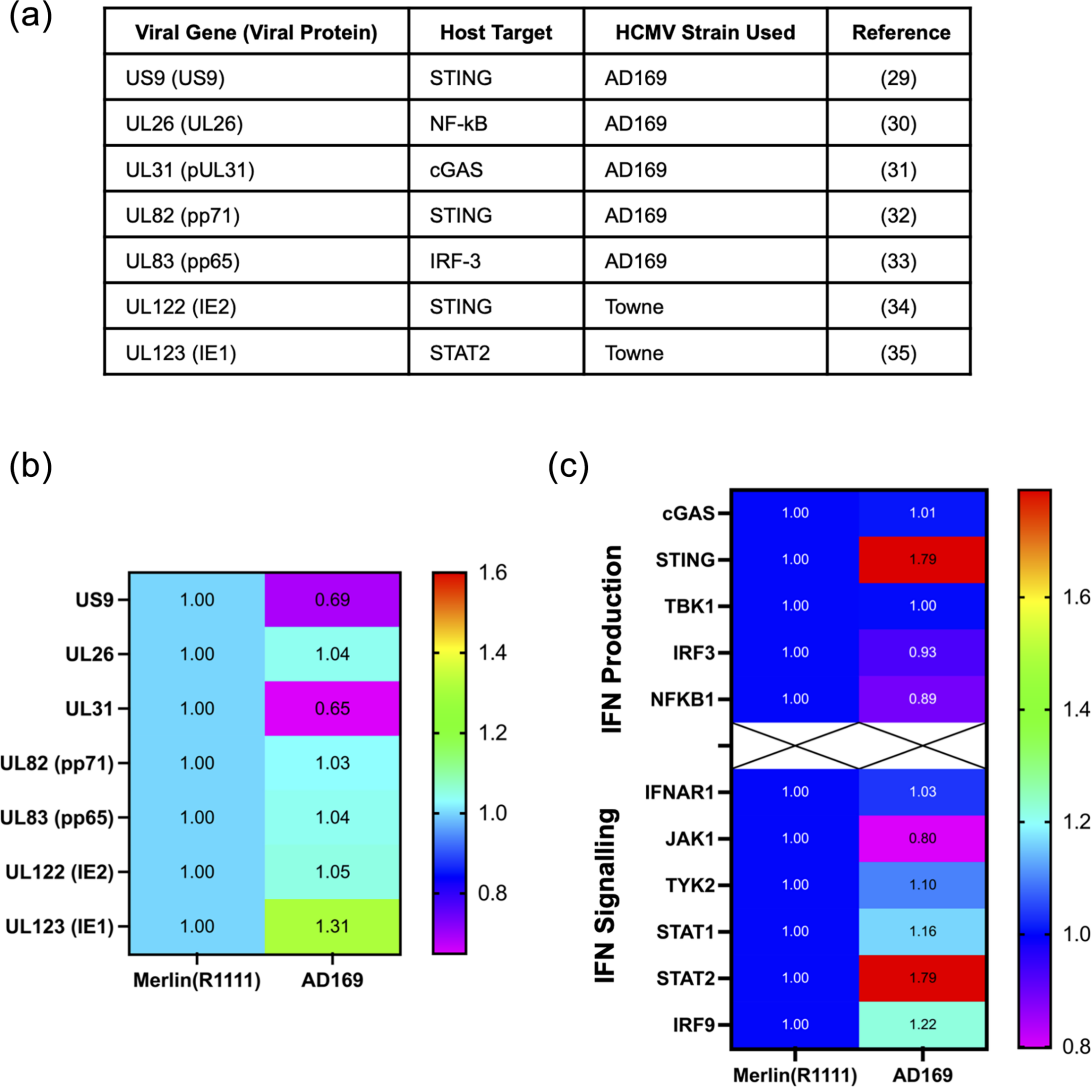
Expression of type I IFN pathway proteins during HCMV infection. (**a**) HCMV type I IFN antagonists, cellular target protein and the strain that was used to characterize viral protein function. (**b**) Heat map of known HCMV IFN antagonist protein expression after 96 h infection with Merlin(R1111) or AD169 in HFFF cells. AD169 expression normalized to Merlin(R1111) expression of 1 (data taken from K.Nightingale *et al.* [22]). (**c**) Heat map of cellular IFN production and IFN signalling protein expression after 96 h infection with Merlin(R1111) or AD169 in HFFF cells. AD169 expression normalized to Merlin(R1111) expression of 1 (from Nightingale *et al.* [22]).

To date, there are seven HCMV proteins produced by both Merlin(R1111) and AD169 reported to be IFN pathway antagonists. It remains unclear if most of these proteins directly interact with the cellular proteins they antagonize (Fig. 1a) [29,35]. As there are no antibodies recognizing many of these HCMV proteins, we could not directly investigate protein expression using standard protein expression assays such as Western blotting. Therefore, we extracted data from published proteomic datasets in which quantitative mass spectrometry was used to assay HCMV protein expression after 96 h infection of HFFT-TERT cells with either Merlin(R1111) or AD169 [22,28]. We investigated any difference in protein expression of the seven HCMV proteins shown in Fig. 1a in AD169-infected cells compared to Merlin-infected cells (Fig. 1b). These data showed no obvious difference in protein expression of UL26, UL82 (pp71), UL83 (pp65) and UL122 (IE2). However, we observed a modest decrease (less than twofold) in US9 expression and a modest increase in UL123 (IE1) expression in AD169-infected cells compared to Merlin(R1111)-infected cells (Fig. 1b).

We then investigated if the cellular proteins antagonized by the HCMV proteins had corresponding differences in expression during infection. Using the same published data set as above (Fig. 1b) [22,28], we extracted the information on expression of proteins involved in type I IFN production and signalling after 96 h of infection with Merlin(R1111) or AD169 in HFFF-TERT cells (Fig. 1c). Modest differences in the expression of several proteins were found (IRF3, NF-κB p50 and JAK1). However, the most notable, albeit modest, differences in protein expression were increased expression of STING and STAT2 in AD169-infected cells when compared to Merlin(R1111)-infected cells. These proteins have been reported to be antagonized by US9 and UL123, respectively (Fig. 1a). As stated above, US9 expression is decreased in AD169 infection compared to Merlin(R1111), which could account for the increase in STING expression, but conversely, UL123 is increased, which would oppose the increase in STAT2 found.

Overall, we observed modest differences in protein expression of many cellular proteins involved in IFN production and IFN signalling (Fig. 1c). It was interesting to note that differences in US9 and UL123 expression correlated with differences in STING and STAT2 expression, although these effects were also modest. However, these data do not exclude the possibility that all of these differences acting together could result in differences in AD169 and Merlin(R1111) interaction with the type I IFN system. Alternatively, the redundancy in expression of multiple IFN antagonists by HCMV could result in no obvious difference in antagonization of HCMV replication by type I IFNs.

### IFN antagonists expressed by different HCMV strains have different protein sequences and similar structures

**Fig. 2.**
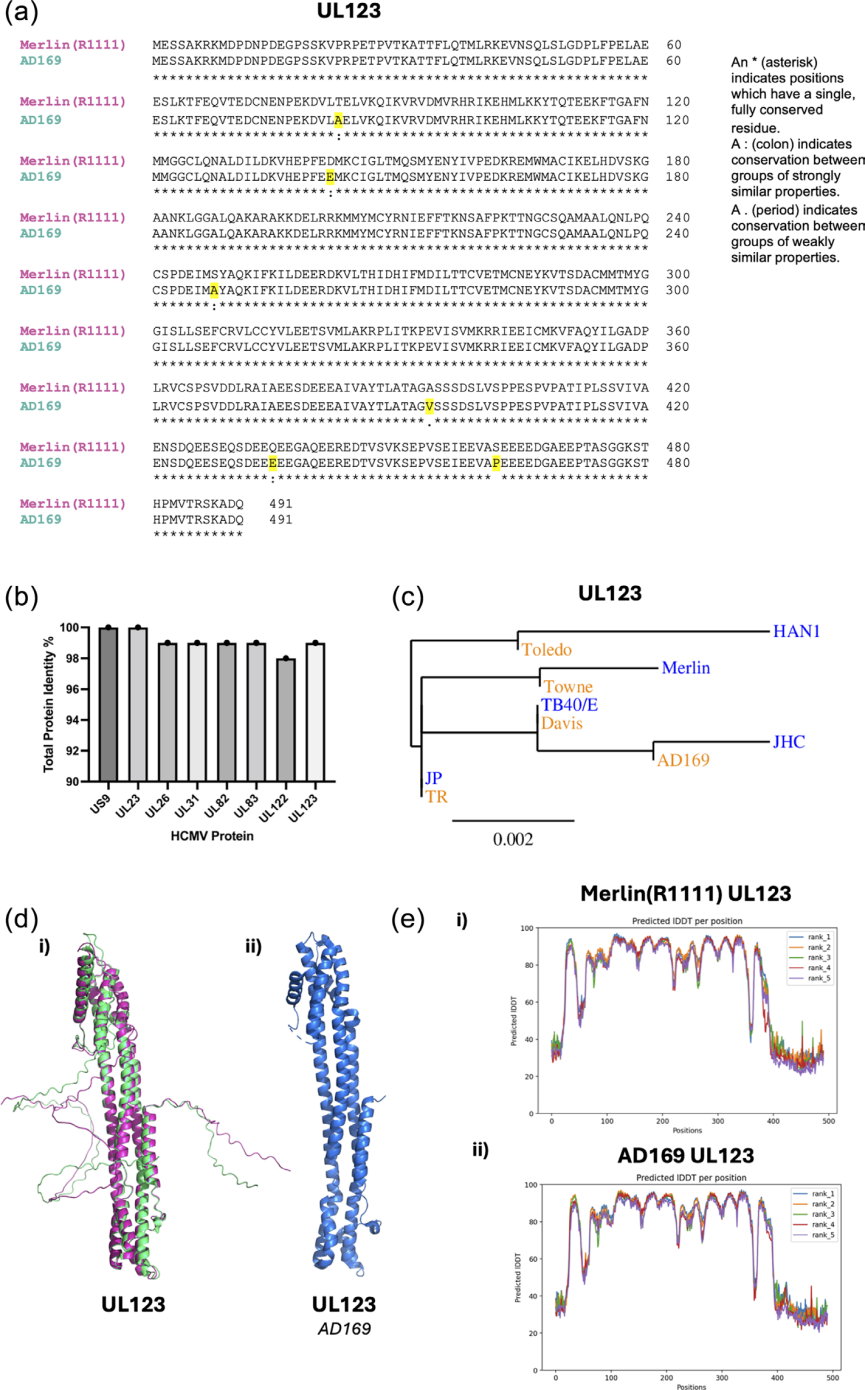
HCMV strain comparison of known type I IFN antagonists. (**a**) Protein sequences were obtained from the NCBI and an alignment of UL123 protein sequences from Merlin(R1111) (pink) and AD169 (green) was created using blast. (**b**) Percentage identity of Merlin(R1111) against AD169 protein sequences of the seven viral proteins listed in Fig. 1a. (**c**) Phylogenetic tree of UL123 from five low (blue) or five high (orange) passage HCMV strains. (**d**) (i) Structures of HCMV UL123 from Merlin(R1111) (pink) and AD169 (green) were created from predicted protein structure using AlphaFold. (ii) Crystal structure of HCMV UL123 from AD169 previously published by Scherer *et al*. [43]. (**e**) Predicted LDDT plots generated by the AlphaFold software during protein structure predictions. (i) Merlin(R1111) UL123, (ii) AD169 UL123. Ranks 1–5 are different models of the same protein prediction. Predicted LDDT value >70 is a high-confidence structural prediction.

 We next investigated if any differences in the protein sequences of the known HCMV IFN antagonists could account for differences in cellular protein expression of type I IFN proteins identified above (Fig. 1c). blast alignments of the seven HCMV proteins shown in Fig. 1a, expressed by Merlin(R1111) and AD169, were performed. Each alignment is shown in panel (i) of Fig. S1A–G (available in the online Supplementary Material). A representative example of an alignment for UL123 is shown in Fig. 2a. In each case, differences in protein sequence were found throughout each protein sequence from different strains. From these alignments, we obtained percentage identity values for each AD169 protein sequence compared to the Merlin(R1111) protein sequence (Fig. 2b). While US9 showed no difference in percentage identity between strains, UL26, UL31, UL82, UL83 and UL123 differed by 1% identity and UL122 by 2% identity. Therefore, there were only modest differences in protein sequence between Merlin(R1111) and AD169 proteins.

However, it remained a possibility that even modest differences in protein sequence could impact protein function. We reasoned that protein sequences important for HCMV replication would be conserved in low-passage strains under immunological pressure *in vivo*, but not in high-passage strains passaged in laboratory settings with no immunological pressure. Therefore, we selected five widely studied high-passage strains and five low-passage strains (the widely studied Merlin and TB40/E strains, plus three selected at random) from the NCBI database and performed ClustralΩ alignment [panel (ii), Fig. S1A–G] and phylogenetic analysis [panel (iii), Fig. S1A–G] of all seven HCMV proteins involved in type I IFN antagonism [36,42]. We found no obvious conservation of mutations across high- or low-passage strains, and phylogenetic trees did not show any obvious evolutionary divergence of protein sequences from low- and high-passage strains. An example of a protein tree (UL123) is shown in Fig. 2c. This suggested that the differences in protein sequences between Merlin(R1111) and AD169 were unlikely to have an obvious impact on protein function.

We wished to investigate whether the combination of mutations throughout the protein sequence of AD169 compared to Merlin(R1111) altered the overall structure of the protein. However, as yet, there is only one full-length atomic resolution structure of any of our HCMV proteins of interest (Fig. 1a); UL123 expressed by AD169 [43]. Therefore, we generated predicted structures of Merlin(R1111) and AD169 proteins using AlphaFold [27] [Figs. 2d(i) and S1A–G(iv)]. We compared the published structure of AD169 UL123 with the structure generated by AlphaFold and found very little obvious difference in the structures of the UL123 proteins [Fig. 2d(ii)]. To further assess the validity of the predicted protein structures generated by AlphaFold, we analysed the predicted long distance difference test (LDDT) of five different models generated per protein for each strain [Fig. 2e(i)and S1A–G(v)]. This allowed us to understand if AlphaFold predicted one or many structures for each protein. We found very little or no variation between the LDDT values between the different models of each protein, indicating that AlphaFold confidently predicted the HCMV protein structures examined here.

The AlphaFold structural predictions of monomers of each viral protein for Merlin(R1111) and AD169 sequences are shown in panel (iv) of Fig. S1A–G. We found no obvious difference in the predicted structure of proteins from different strains. However, the functional domains related to IFN antagonistic functions of HCMV proteins have yet to be characterized for the majority of these proteins, including domains required for protein–protein interaction with known, or as yet unknown, protein interactors. Therefore, it remained a possibility that a small number of amino acid changes within a critical domain could alter protein function. However, our overall analysis of proteins from Merlin(R1111) and AD169 suggested that differences in the expression of cellular proteins involved in the type I IFN response were unlikely to be due to differences in the sequence and structure of viral protein antagonists of type I IFN encoded by different HCMV strains.

### IRF3 expression in HCMV infection was MOI-dependent

**Fig. 3.**
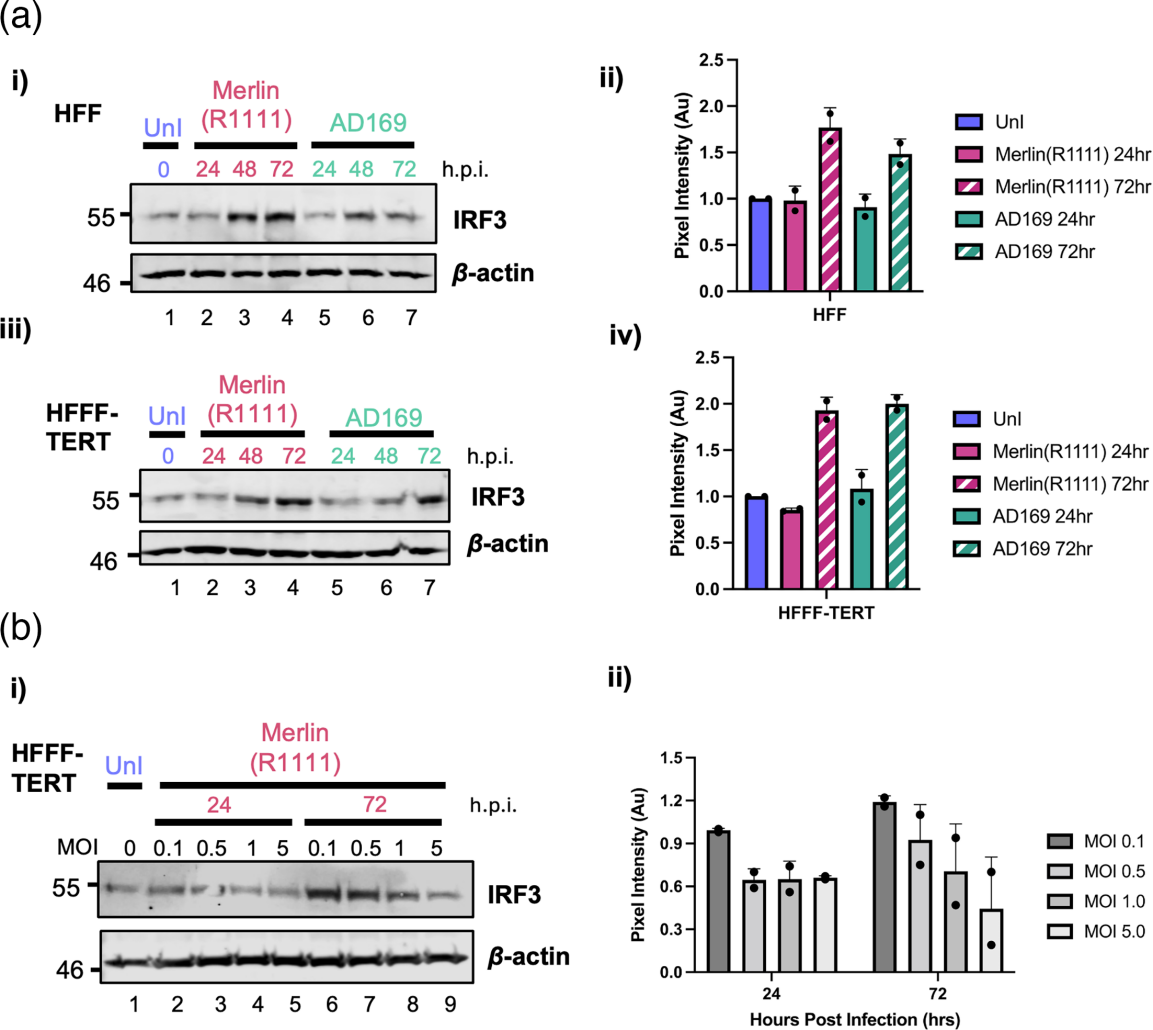
Western blotting of HFF and HFFF-TERT cells infected with Merlin(R1111) and AD169. (**a**) HFF (**i**) and HFFF-TERT (iii) cells were infected with Merlin(R1111) or AD169 at an MOI of 1. Cell lysates were prepared for Western blotting from uninfected cells at 0 h.p.i. and from HFF and HFFF-TERT cells infected with Merlin(R1111) and AD169 at 24, 48 and 72 h.p.i. Proteins recognized by the antibodies are indicated to the left of each panel. Positions of molecular mass markers (kDa) are indicated on the outer side of each respective blot. Pixel intensity was measured using ImageJ and is shown for HFF (ii) and HFFF-TERT (iv). Blocks and error bars in (ii) and (iv) represent the mean and range, respectively, from two independent experiments. (**b**) HFFF-TERT (iii) cells were infected with Merlin(R1111) at an MOI of 0.1–5. Cell lysates were prepared for Western blotting from uninfected cells at 0 h.p.i. and from HFFF-TERT cells infected with Merlin(R1111) at 24 and 72 h.p.i. Proteins recognized by the antibodies are indicated to the left of each panel. Positions of molecular mass markers (kDa) are indicated on the outer side of each respective blot. Pixel intensity was measured using ImageJ and is shown for HFFF-TERT (ii). Blocks and error bars in (ii) represent the mean and range, respectively, from two independent experiments.

In the datasets investigated above (Fig. 1c, d), we also considered those proteins with no obvious differences in protein expression, for example, IRF3. It was previously reported using quantitative mass spectrometry and Western blotting in Merlin(R1111)-infected HFFF-TERT cells that IRF3 expression decreased over time [28]. Therefore, we thought it possible that differences in the rate of IRF3 decrease over time in Merlin(R1111)- and AD169-infected cells could have influenced control of IFN during infection. However, our previous observations of IRF3 expression in Merlin(R1111)-infected HFF cells using Western blotting had indicated that IRF3 expression increased, not decreased, over time [23]. To understand IRF3 expression over time and to reconcile differences in reported IRF3 expression, we chose to investigate IRF3 expression in both HFF and HFFF-TERT cells in parallel. HFF and HFFF-TERT cells were infected with Merlin(R1111) or AD169, and IRF3 expression was assayed using Western blotting (Fig. 3a). IRF3 expression increased over time, 24 to 72 h post-infection (h.p.i.), in all experiments [Fig. 3a(i, iii)].

 We next investigated the possible conflicting observations of IRF3 expression in HFFF-TERT cells [23,28]. It was possible that differences in our observations and those previously reported [23,28] were due to differences in experimental procedure between studies. In the previous studies, HCMV infection was carried out with serum starvation, plus the presence of dexamethasone, and using an increased MOI (23,28). Therefore, we set out to examine whether serum starvation, dexamethasone or MOI had a role in increasing or decreasing IRF3 expression in HFFF-TERT cells upon HCMV infection.

IRF3 expression in HFFF-TERT cells was assayed under a range of conditions using Western blotting (Figs 3b and S2). Serum starvation had no obvious effect on IRF3 expression (Fig. S2A), whereas serum starvation in combination with dexamethasone treatment appeared to decrease global expression of protein, as both IRF3 and *β*-actin expression decreased (Fig. S2B). However, when differences in MOI were tested, we found that high MOI resulted in less IRF3 expression compared to low MOI [Fig. 3b(i, ii)]. Overall, we conclude that differences in IRF3 expression observed between studies were likely due to the use of high MOIs in those studies, wherein the use of high MOI resulted in loss of IRF3 expression over time.

### Differences in cellular protein expression were not associated with differences in IFN or HCMV production

**Fig. 4.**
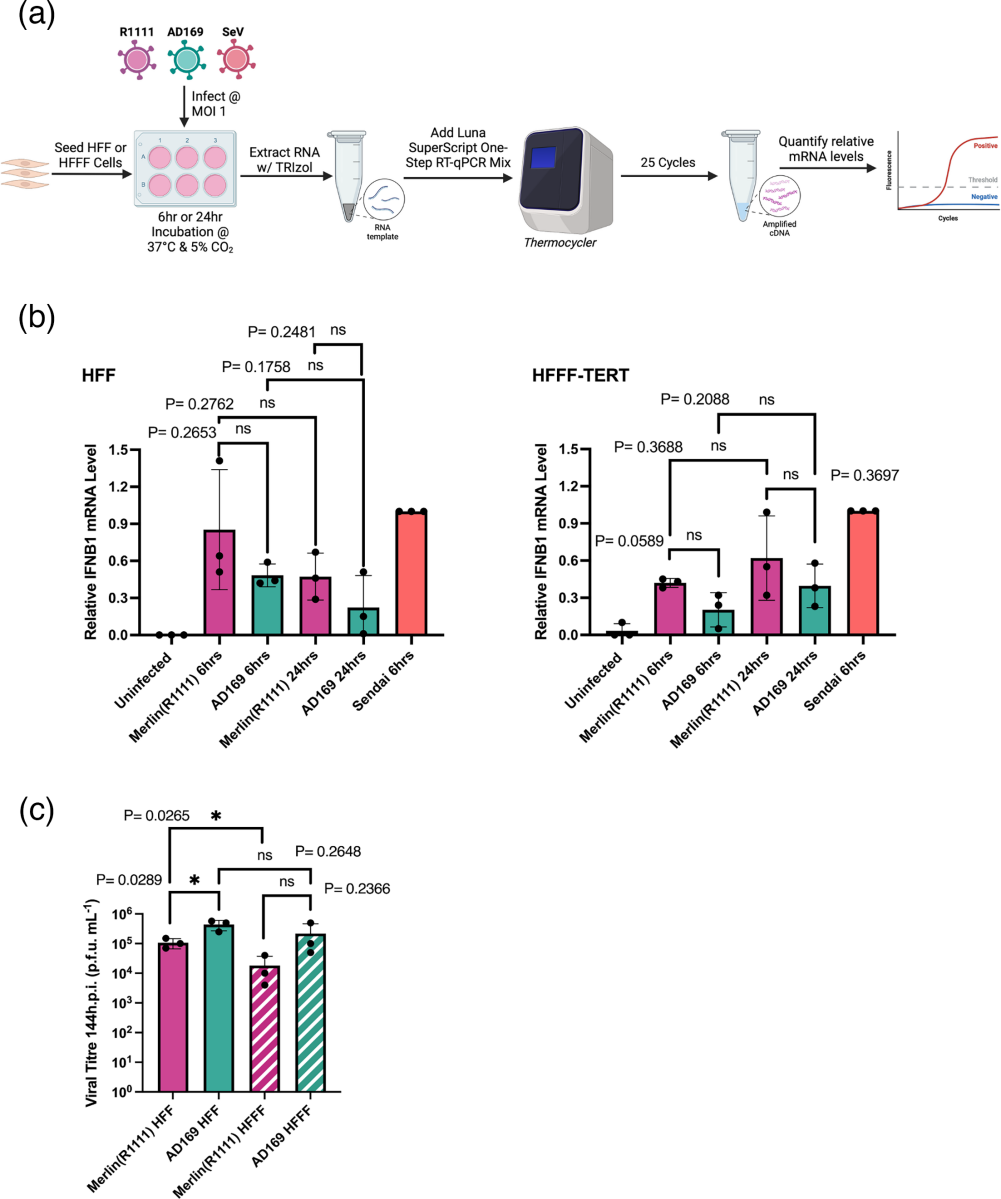
IFN-*β* production during HCMV infection and viral replication assay. (**a**) Diagram of reverse transcriptase qPCR workflow. HFF or HFFF-TERT cells were infected with Merlin(R1111) or AD169 at an MOI of 1 or with SeV at a 1:50 dilution. Cell lysates were collected after 6 h.p.i., and RNA extraction was performed using TRIzol. IFN-*β* and GAPDH RNAs were quantified using reverse transcriptase qPCR. Created in BioRender. Latham, K. (2006) http://BioRender.com/dcorx8o (**b**) Quantitative analysis of reverse transcriptase qPCR, all IFN-*β* values were normalized to their respective GAPDH values. Results are shown for both Merlin(R1111) (pink) and AD169 (green) infection on HFF or HFFF-TERT cells. Data are representative of one biological replicate from three independent experiments: each normalized to their respective SeV (red) values. (**c**) Replication of Merlin(R1111) and AD169 in HFF and HFFF-TERT cells. HFF and HFFF-TERT cells were infected with Merlin(R1111) or AD169 at an MOI of 1. Virus was harvested at 144 h post-infection, and viral titre was determined by titration of virus supernatant on HFF cells. In (**b**) and (**c**), blocks and error bars represent the mean and sd, respectively, of three independent experiments. Data were analysed using a two-tailed unpaired t-test; *P*-values less than or equal to 0.05 (*), 0.01 (**) or 0.001 (***) were considered statistically significant.

 Finally, we determined if there were functional differences in IFN and HCMV production between our two HCMV strains. We infected HFF or HFFF-TERT cells with Merlin(R1111) and AD169 at MOI 1 and examined IFN production using reverse transcriptase qPCR, compared to cells infected with a virus (Sendai virus) known to robustly stimulate type I IFN production (Fig. 4a, b). Transcription of the IFN-*β* gene was stimulated by both HCMV strains early in replication (6 h.p.i.). However, later in HCMV replication (24 h.p.i.), there was a modest decrease in IFN-*β* gene expression in HFF cells and a modest increase in IFN-*β* gene expression in HFFF-TERT cells (Fig. 4b). However, none of these changes in IFN-*β* gene expression were statistically significant (Fig. 4b).

 Production of infectious HCMV from infected cells was assayed using virus replication assays. We infected HFF or HFFF-TERT cells with Merlin(R1111) and AD169 and investigated HCMV titre using plaque assays (Fig. 4c). In both cell lines, AD169-infected cells produced more infectious virus than Merlin(R1111)-infected cells, but this was only moderately statistically significant in HFF cells (Fig. 4c). Therefore, these data indicated no significant differences in the ability of HCMV strains to produce IFN-*β* or to replicate. Additionally, there were no obvious differences in phenotypes observed in HFF and HFFF-TERT cells.

## Discussion

 Overall, this study presents interesting null results that extend our own earlier investigations and provide data that point toward ways in which the study of HCMV interaction with the type I IFN system can be refined in the future.

 Our initial hypothesis in this study was that the differences in HCMV strains’ ability to regulate production of ISGs ZAP-S and MxA resulted in our previous observation: that the low-passage wild-type strain Merlin(R1111) could effectively inhibit the type I IFN response, whereas the high-passage laboratory HCMV strain AD169 could not [23]. The data presented here did not support this. We found that there was no obvious statistical difference in the production of IFN-*β* or the production of infectious HCMV when Merlin(R1111) was compared to AD169. This was supported further by our analysis (data not shown) of quantitative mass spectrometry datasets of the expression of 200 ISGs expressed in Merlin(R1111)- and AD169-infected cells, wherein there was little or no difference in ISG expression, including MxA and ZAP-S.

 The lack of obvious differences in IFN-*β* and HCMV production between strains was associated with differences in the expression of known HCMV IFN antagonists and cellular proteins associated with type I IFN production and signalling. However, we recognize that all observed differences were very modest (less than twofold) and there was not one obvious candidate protein that could have obviously influenced the interaction of HCMV with the IFN system. We speculate that all the modest differences in viral and cellular protein expression we observe could result in no differences in Merlin(R1111) and AD169 interaction with the type I IFN system and/or the redundancy in expression of multiple IFN antagonists encoded by HCMV would result in no obvious difference in antagonization of HCMV replication by type I IFNs between HCMV strains. It must be noted that the present study is somewhat limited, as very few large-scale proteomic datasets examining HCMV interaction with the IFN system are available. These should be developed in future work. A comparison of different datasets will address any concerns around the reproducibility of data between studies, especially in studies such as ours, where there are only modest differences in protein expression.

 Importantly, the data presented here do not explain our previous observations that ISGs MxA and ZAP-S were robustly expressed in AD169-infected cells, but not Merlin(R1111)-infected cells [23]. Thus, we speculate that MxA and ZAP-S expression was influenced directly by proteins expressed by Merlin(R1111), but not AD169. Alternatively, ZAP-S expression was influenced by proteins expressed by both strains, but one strain contains a mutational change that prevents its proteins from interacting with the specific aforementioned ISGs. It remains unknown what these proteins might be. As we and others have demonstrated that ZAP-S is an inhibitor of HCMV replication [23,44], HCMV may possess a protein whose function is to specifically inhibit ZAP-S expression. It remains unknown if MxA has anti-HCMV activity.

 An interesting finding in our study was that expression of IRF3 was dependent on the MOI used in our experiments. We think it is possible that at low MOI, few cells are infected and the type I IFN response stimulates IRF3 expression. However, at high MOI, all cells in the culture are infected, and antagonism of the type I IFN system by HCMV inhibits IRF3 expression. Conversely, it is also possible that during low MOI infections, IRF3 expression is upregulated by uninfected bystander cells; hence, we observe an overall increase in IRF3 expression in the total cell population in our experiments. These possibilities have yet to be experimentally examined. We do not believe that high MOI infection leads to cellular toxicity that affects protein expression, as we see no loss of infected cells in our high MOI-infected cell culture (data not shown) and we find no loss of cellular protein expression in infected cells (*β*-actin expression). We noted that the two aforementioned studies of IRF3 expressions used different antibodies for IRF3 Western blotting; the HFFF-TERT cell study used a polyclonal antibody, whereas the HFF cell study used a monoclonal antibody [23,28]. However, both antibodies used within the studies were designed to detect endogenous levels of total IRF3 protein and, therefore, are unlikely to be the cause of the different expression patterns seen between studies [23,28]. Additionally, there may be MOI-dependent effects on the expression of other proteins involved in the type I IFN system, other than IRF3. Thus, expression of cellular proteins analysed in Fig. 1 may reflect influences on protein expression by the presence of IFN antagonists and MOI-dependent effects.

Our observation of MOI-dependent IRF3 expression impacts our current study and future studies. All quantitative mass spectrometry data analysed here were taken from cells infected at high MOI (MOI 10) [22,28]. Therefore, in the current study, our analysis of viral and protein expression could reflect cellular protein expression influenced by HCMV IFN antagonists and MOI-dependent effects. Re-evaluation of the mass spectrometry data using low MOI in future experiments may yield further insights into the antagonism of the type I IFN system by HCMV. Looking ahead, it may be wise to include MOI as a factor in any future experiments studying IRF3 and other factors associated with the type I IFN response in HCMV-infected cells. Plus, based on our observations, it is possible that the study of HCMV interaction with the type I IFN system will be similar in adult and foetal fibroblast cells.

## Supplementary material

10.1099/acmi.0.001104.v3 Uncited Supplementary Material 1.

## Abbreviations

DMEM: Dulbecco’s Modified Eagle Medium
GAPDH: glyceraldehyde-3-phosphate dehydrogenase
HCMV: human cytomegalovirus
HFF: human foreskin fibroblast
HFFF-TERT: human foetal foreskin fibroblast
h.p.i.: h post-infection
IRF3: IFN regulatory factor 3
ISGs: IFN-stimulated genes
JAK: Janus protein tyrosine kinase
LDDT: long distance difference test
MOI: multiplicity of infection
NCBI: National Center for Biotechnology Information
NF-κB: nuclear factor-κB
qPCR: quantitative PCR
SeV: Sendai virus
STAT: signal transducers and activators of transcription
TBS-T: tris-buffered saline with Tween 20
