# Supplementary material for: Human cytomegalovirus strain-specific differences in protein expression of type I IFN pathway proteins do not impact virus replication
Source: Access Microbiol. 2026 Feb 19;8(2):001104.v3. doi: 10.1099/acmi.0.001104.v3 (PMC12917765; doi:10.1099/acmi.0.001104.v3)

Human Cytomegalovirus Strain Specific Differences in Protein Expression of Type I IFN Pathway Proteins Do Not Impact Virus Replication.

Katie A Latham[a], Timothy K Soh[b,c,d,e], Richard J Stanton[f], Jens B Bosse[b,c,d,e], Steve Goodbourn[a] & Blair L Strang[a]

Institute of Infection & Immunity, St George's School of Health and Medical Sciences, City St George's University of London, UK[a]. Centre for Structural Systems Biology, Hamburg, Germany[b]. Hannover Medical School, Institute of Virology, Hannover, Germany[c]. Cluster of Excellence RESIST (EXC 2155), Hannover Medical School, Hannover, Germany[d]. Leibniz Institute of Virology (LIV), Hamburg, Germany[e]. Centre for Structural Systems Biology, Hamburg, Germany[b]. Division of Infection and Immunity, Cardiff University School of Medicine, UK[f].

Supplementary Data 1

(A) HCMV protein US9. (B) HCMV protein UL23. (C) HCMV protein UL26. (D) HCMV protein UL31. (E) HCMV protein UL82. (F) HCMV protein UL83. (G) HCMV protein UL122. (H) HCMV protein U123. (i) Two strain alignment using NCBI BLAST, Merlin(R1111) (pink) and AD169 (green). Mutations present in AD169 are highlighted in yellow. (ii) Phylogenetic tree of 10 HCMV strains, low passage strains in blue and high passage strains in orange. (iii) Ten strain alignment using Clustralω, low passage strains in blue and high passage strains in orange. Mutations differing to Merlin(R1111) sequence are highlighted in yellow. Mutations are characterised by the key: * (asterisk) indicates fully conserved residues, : (colon) indicates strongly similar properties, . (period) indicates conversation of weakly similar properties and empty space indicates no similarity. (iv) Alphafold monomer predictions; structured aligned with Merlin(R1111) in pink and AD169 in green. (v) Predicted IDDT plots for five different models for each predicted protein structures. Predicted IDDT value >70 is a high confidence structural prediction.

Supplementary Data 2

(A) (i) Time course of treatment and infection. (ii) HFFF-TERT cells were cultured in 0% or 10% FBS and with or without Dexamethasone for 24 hours prior to infection. Cells were infected with Merlin(R1111) or AD169 at an MOI of 1. Cell lysates were prepared for western blotting from uninfected cell at 0h.p.i., and from HFF and HFFF-TERT cells infected with Merlin(R1111) and AD169 at 96 h.p.i. Proteins recognised by the antibodies are indicated to the left of each panel. Positions of molecular mass markers (kDa) are indicated on the outer side or each respective blot. (B) (i) Time course of treatment and infection. (ii) HFFF-TERT cells were cultured in 0% or 10% FBS for 24 hours prior to infection. Cells were infected with Merlin(R1111) or AD169 at an MOI of 1. Cell lysates were prepared for western blotting from uninfected cell at 0h.p.i., and from HFF and HFFF-TERT cells infected with Merlin(R1111) and AD169 at 96 h.p.i. Proteins recognised

by the antibodies are indicated to the left of each panel. Positions of molecular mass markers (kDa) are indicated on the outer side or each respective blot.

Supplementary Figure 1

A- US9

i)

```
Merlin      MILWSPSTCSFFWHWCLIAVSVLSSRSKESLRLSWSSDESSASSSSRICPLSNSKSVRLP   60
AD169       MILWSPSTCSFFWHWCLIAVSVLSSRSKESLRLSWSSDESSASSSSRICPLSNSKSVRLP   60
            ************************************************************

Merlin      QYPRGFGDVSGYRVSSSVSECYVQHGVLVAAWLVRGNFSDTAPRAYGTWGNERSATHFKV   120
AD169       QYPRGFGDVSGYRVSSSVSECYVQHGVLVAAWLVRGNFSDTAPRAYGTWGNERSATHFKV   120
            ************************************************************

Merlin      GAPQLENDGALRYETELPQVDARLSYVMLTVYPCSACNRSVLHCRPASRLPWLPLRVTPS   180
AD169       GAPQLENDGALRYETELPQVDARLSYVMLTVYPCSACNRSVLHCRPASRLPWLPLRVTPS   180
            ************************************************************

Merlin      DLERLFAERRYLTFLYVVLVQFVKHVALFSFGVQVACCVYLRWIRPWVRGRHRATGRTSR   240
AD169       DLERLFAERRYLTFLYVVLVQFVKHVALFSFGVQVACCVYLRWIRPWVRGRHRATGRTSR   240
            ************************************************************

Merlin      EEEAKDD      247
AD169       EEEAKDD      247
            *******
```

ii)

```
Merlin      MILWSPSTCSFFWHWCLIAVSVLSSRSKESLRLSWSSDESSASSSSRICPLSNSKSVRLP   60
TB40/E      MILWSPSTCSFFWHWCLIAVSVLSSRSKESLRLSWSSDESSASSSSRICPLSNSKSVRLP   60
JP          MILWSPSTCSFFWHWCLIAVSVLSSRSKESLRLSWSSDESSASSSSRICPLSDSKSVRLP   60
JHC         MILWSPSTCSFFWHWCLIAVSVLSNRSKQSLRLSWSNDESSASSSSRICPLSNSKSVRLP   60
HAN1        MILWSPSTCSFFWHWCLIAVSVLSSRSKESLRLSWSSDESSASSSSRICPLSDSKSVRLP   60
AD169       MILWSPSTCSFFWHWCLIAVSVLSSRSKESLRLSWSSDESSASSSSRICPLSNSKSVRLP   60
Toledo      MILWSPSTCSFFWHWCLIAVSVLSSRSKESLRLSWSSDESSASSSSRICPLSDSKSVRLP   60
Davis       MILWSPSTCSFFWHWCLIAVSVLSSRSKESLRLSWSSDESSASSSSRICPLSDSKSVRLP   60
Towne       MILWSPSTCSFFWHWCLIAVSVLSSRSKESLRLSWSSDESSASSSSRICPLSDSKSVRLP   60
TR          MILWSPSTCSFFWHWCLIAVSVLSSRSKESLRLSWSSDESSASSSSRICPLSNSKSVRLP   60
            ************************.***:*******.**************:********

Merlin      QYPRGFGDVSGYRVSSSVSECYVQHGVLVAAWLVRGNFSDTAPRAYGTWGNERSATHFKV   120
TB40/E      QYPRGFGDVSGYRVSSSVSECYVQHGVLVAAWLVRGNFSDTAPRAYGTWGNERSATHFKV   120
JP          QYPRGFGDVSGYRVSSSVSECYVQHGVLVAAWLVRGNFSDTAPRAYGTWGNERSATHFKV   120
JHC         QYPRGFGDVSGYRVSSSVSECYVQHGVLVAAWLVRGNFSDTAPRAYGTWGNERSATHFKV   120
HAN1        QYPRGFGDVSGYRVSSSVSECYVQHGVLVAAWLVRGNFSDTAPRAYGTWGNERSATHFKV   120
AD169       QYPRGFGDVSGYRVSSSVSECYVQHGVLVAAWLVRGNFSDTAPRAYGTWGNERSATHFKV   120
Toledo      QYPRGFGDVSGYRVSSSVSECYVQHGVLVAAWLVRGNFSDTAPRAYGTWGNERSATHFKV   120
Davis       QYPRGFGDVSGYRVSSSVSECYVQHGVLVAAWLVRGNFSDTAPRAYGTWGNERSATHFKV   120
Towne       QYPRGFGDVSGYRVSSSVSECYVQHGVLVAAWLVRGNFSDTAPRAYGTWGNERSATHFKV   120
TR          QYPRGFGDVSGYRVSSSVSECYVQHGVLVAAWLVHGNFSDTAPRAYGTWGNERSATHFKV   120
            **********************************:*************************

Merlin      GAPQLENDGALRYETELPQVDARLSYVMLTVYPCSACNRSVLHCRPASRLPWLPLRVTPS   180
TB40/E      GAPQLENDGALRYETELPQVDARLSYVMLTVYPCSACNRSVLHCRPASRLPWLPLRVTPS   180
JP          GAPQLENDGALRYETELPQVDARLSYVMLTVYPCSACNRSVLHCRPASRLPWLPLRATPS   180
JHC         GAPQLENDGALRYETELPQVDARLSYVMLTVYPCSACNRSVLHCRPASRLPWLPLRVTPS   180
HAN1        GAPQLENDGALRYETELPQVDARLSYVMLTVYPCSACNRSVLHCRPASRLPWLPLRATPS   180
AD169       GAPQLENDGALRYETELPQVDARLSYVMLTVYPCSACNRSVLHCRPASRLPWLPLRVTPS   180
Toledo      GAPQLENDGALRYETELPQVDARLSYVMLTVYPCSACNRSVLHCRPASRLPWLPLRATPS   180
Davis       GAPQLENDGALRYETELPQVDARLSYVMLTVYPCSACNRSVLHCRPASRLPWLPLRATPS   180
Towne       GAPQLENDGALRYETELPQVDARLSYVMLTVYPCSACNRSVLHCRPASRLPWLPLRATPS   180
TR          GAPQLENDGALRYETELPQVDARLSYVMLTVYPCSACNRSVLHCRPASRLPWLPLRVTPS   180
            ********************************************************.***

Merlin      DLERLFAERRYLTFLYVVLVQFVKHVALFSFGVQVACCVYLRWIPWVRGRHRATGRTSR   240
TB40/E      DLERLFAERRYLTFLYVVLVQFVKHVALFSFGVQVACCVYLRWIPWVRGRHRATGRTSR   240
JP          DLERLFAERRYLTFLYVVLVQFVKHVALFSFGVQVACCVYLRWIPWVRGRHRATGRTSR   240
JHC         DLERLFAERRYLTFLYVVLVQFVKHVALFSFGVQVACCVYLRWIPWVRGRHRATGRTSR   240
HAN1        DLERLFAERRYLTFLYVVLVQFVKHVALFSFGVQVACCVYLRWIPWVRGRHRATGRTSR   240
AD169       DLERLFAERRYLTFLYVVLVQFVKHVALFSFGVQVACCVYLRWIPWVRGRHRATGRTSR   240
Toledo      DLERLFAERRYLTFLYVVLVQFVKHVALFSFGVQVACCVYLRWIPWVRGRG--------   232
Davis       DLERLFAERRYLTFLYVVLVQFVKHVALFSFGVQVACCVYLRWIPWVRGRHRATGRTSR   240
Towne       DLERLFAERRYLKFLYVVLVQFVKHVALFSFGVQVACCVYLRWIPWVRGRG--------   232
TR          DLERLFAERRYLTFLYVVLVQFVKHVALFSFGVQVAYCVYLRWIPWVRGRHRATGRTSR   240
            ************.*********************** **************

Merlin      EEEAKDD   247
TB40/E      EEEAKDD   247
JP          EEEAKDD   247
JHC         EEEAKDD   247
HAN1        EEEAKDD   247
AD169       EEEAKDD   247
Toledo      -------   232
Davis       EEEAKDD   247
Towne       -------   232
TR          EEEAKDD   247
```

iii)

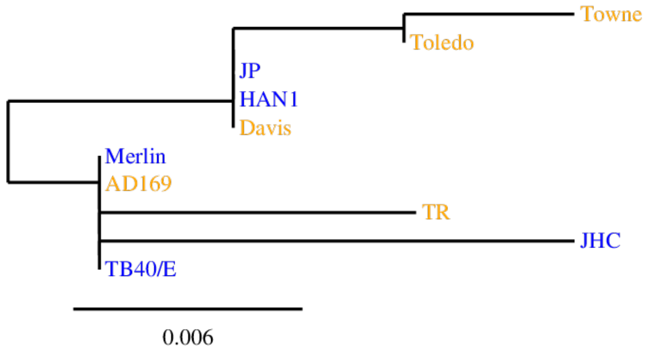
Towne
Toledo
JP
HAN1
Davis
Merlin
AD169
TR
JHC
TB40/E
0.006

iv)

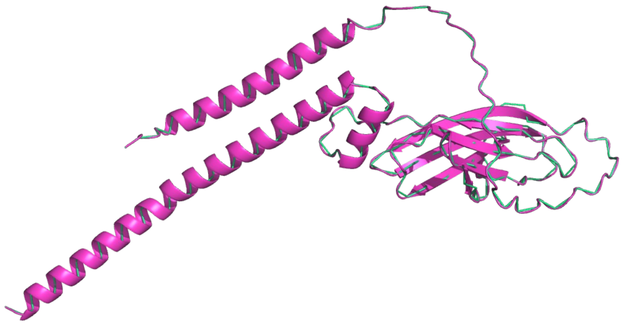

v)

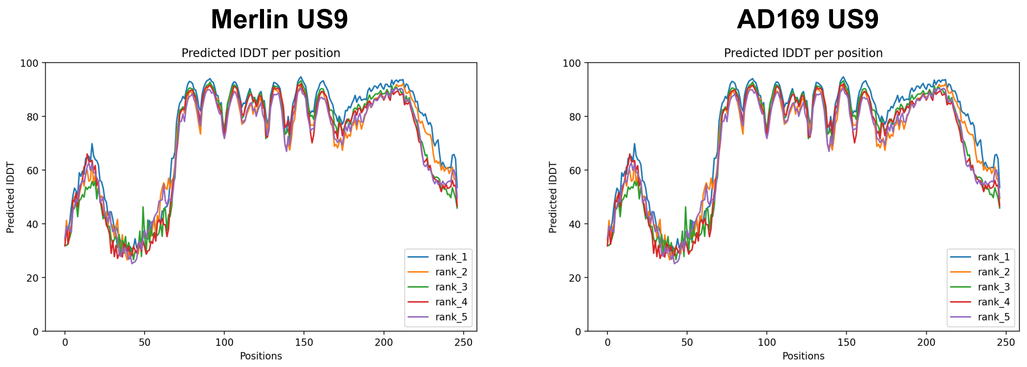
Merlin US9
Predicted lDDT per position
Predicted lDDT
Positions
rank_1
rank_2
rank_3
rank_4
rank_5
AD169 US9
Predicted lDDT per position
Predicted lDDT
Positions
rank_1
rank_2
rank_3
rank_4
rank_5

B- UL26

i)

```
Merlin    MTSRRAPDGGLNLDDFMRRQRGRHLDLPYPRGYTLFVCDVEETILTPRDVEYWKLLVVTQ   60
AD169     MTSRRAPDGGLNLDDFMRRQRGRHLDLPYPRGYTLFVCDVEETILTPRDVEYWKLLVVTQ   60
          ************************************************************

Merlin    GQLRVIGTIGLANLFSWDRSVAGVAADGSVLCYEISRENFVVRAADSLPQLLERGLLHSY   120
AD169     GQLRVIGTIGLANLFSWDRSVAGVAADGSVLCYEISRENFVVRAADSLPQLLERGLLHSY   120
          ************************************************************

Merlin    FEDVERAAQGRLRHGNRSGLRRDADGQVIRESACYVSRALLRHRVTPGKQEITDAMFEAG   180
AD169     FEDVERAAQGRLRHGNRSGLRRDADGQVIRESACYVSRVLLRHRVTPGKQEITDAMFEAG   180
          **************************************.*********************

Merlin    NVPSALLP   188
AD169     NVPSALLP   188
          ********
```

ii)

```
Merlin      MTSRRAPDGGLNLDDFMRRQRGRHLDLPYPRGYTLFVCDVEETILTPRDVEYWKLLVVTQ    60
TB40/E      MTSRRAPDGGLNLDDFMRRQRGRHLDLPYPRGYTLFVCDVEETILTPRDVEYWKLLVVTQ    60
JP          MTSRRAPDGGLNLDDFMRRQRGRHLDLPYPRGYTLFVCDVEETILTPRDVEYWKLLVVTQ    60
JHC         MTSRRVPDGGLNLDDFMRRQRGRHLDLPYPRGYTLFVCDVEETILTPRDVEYWKLLVVTQ    60
HAN1        MTSRRAPDGGLNLDDFMRRQRGRHLDLPYPRGYTLFVCDVEETILTPRDVEYWKLLVVTQ    60
AD169       MTSRRAPDGGLNLDDFMRRQRGRHLDLPYPRGYTLFVCDVEETILTPRDVEYWKLLVVTQ    60
Toledo      MTSRRAPDGGLNLDDFMRRQRGRHLDLPYPRGYTLFVCDVEETILTPRDVEYWKLLVVTQ    60
Davis       MTSRRAPDGGLNLDDFMRRQRGRHLDLPYPRGYTLFVCDVEETILTPRDVEYWKLLVVTQ    60
Towne       MTSRRAPDGGLNLDDFMRRQRGRHLDLPYPRGYTLFVCDVEETILTPRDVEYWKLLVVTQ    60
TR          MTSRRAPDGGLNLDDFMRRQRGRHLDLPYPRGYTLFVCDVEETILTPRDVEYWKLLVVTQ    60
            *****.******************************************************

Merlin      GQLRVIGTIGLANLFSWDRSVAGVAADGSVLCYEISRENFVVRAADSLPQLLERGLLHSY    120
TB40/E      GQLRVIGTIGLANLFSWDRSVAGVAADGSVLCYEISRENFVVRAADSLPQLLERGLLHSY    120
JP          GQLRVIGTIGLANLFSWDRSVAGVAADGSVLCYEISRENFVVRAADSLPQLLERGLLHSY    120
JHC         GQLRVIGTIGLANLFSWDRSVAGVAADGSVLCYEISRENFVVRAADSLPQLLERGLLHSY    120
HAN1        GQLRVIGTIGLANLFSWDRSVAGVAADGSVLCYEISRENFVVRAADSLPQLLERGLLHSY    120
AD169       GQLRVIGTIGLANLFSWDRSVAGVAADGSVLCYEISRENFVVRAADSLPQLLERGLLHSY    120
Toledo      GQLRVIGTIGLANLFSWDRSVAGVAADGSVLCYEISRENFVVRAADSLPQLLERGLLHSY    120
Davis       GQLRVIGTIGLANLFSWDRSVAGVAADGSVLCYEISRENFVVRAADSLPQLLERGLLHSY    120
Towne       GQLRVIGTIGLANLFSWDRSVAGVAADGSVLCYEISRENFVVRAADSLPQLLERGLLHSY    120
TR          GQLRVIGTIGLANLFSWDRSVAGVAADGSVLCYEISRENFVVRAADSLPQLLERGLLHSY    120
            ************************************************************

Merlin      FEDVERAAQGRLRHGNRSGLRRDADGQVIRESACYVSRALLRHRVTPGKQEITDAMFEAG    180
TB40/E      FEDVERAAQGRLRHGNRSGLRRDADGQVIRESACYVSRALLRHRVTPGKQEITDAMFEAG    180
JP          FEDVERAAQGRLRHGNRSGLRRDADGQVIRESACYVSRALLRHRVTPGKQEITDAMFEAG    180
JHC         FEDVERAAQGRLRHGNRSGLRRDADGQVIRESACYVSRALLRHRVTPGKQEITDAMFEAG    180
HAN1        FEDVERAAQGRLRHGNRSGLRRDADGQVIRESACYVSRALLRHRVTPGKQEITDAMFEAG    180
AD169       FEDVERAAQGRLRHGNRSGLRRDADGQVIRESACYVSRVLLRHRVTPGKQEITDAMFEAG    180
Toledo      FEDVERAAQGRLRHGNRSGLRRDADGQVIRESACYVSRALLRHRVTPGKQEITDAMFEAG    180
Davis       FEDVERAAQGRLRHGNRSGLRRDADGQVIRESAWYVSRALLRHRVTPGKQEITDAMFEAG    180
Towne       FEDVERAAQGRLRHGKRSGLRRDADGQVIRESACYVSRALLRHRVTPGKQEITDAMFEAG    180
TR          FEDVERAAQGRLRHGNRSGLRRDADGQVIRESACYVSRALLRHRVTPGKQEITDAMFEAG    180
            ***************:***************** ****.*********************

Merlin      NVPSALLP                                                        188
TB40/E      NVPSALLP                                                        188
JP          NVPSALLP                                                        188
JHC         NVPSALLP                                                        188
HAN1        NVPSALLP                                                        188
AD169       NVPSALLP                                                        188
Toledo      NVPSALLP                                                        188
Davis       NVPSALLP                                                        188
Towne       NVPSALLP                                                        188
TR          NVPSALLP                                                        188
            ********
```

iii)

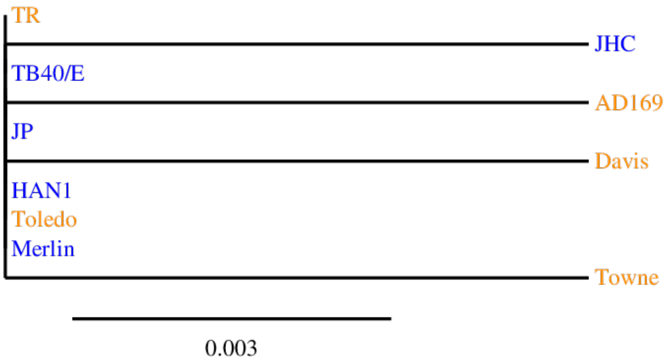
TR
JHC
TB40/E
AD169
JP
Davis
HAN1
Toledo
Merlin
Towne
0.003

iv)

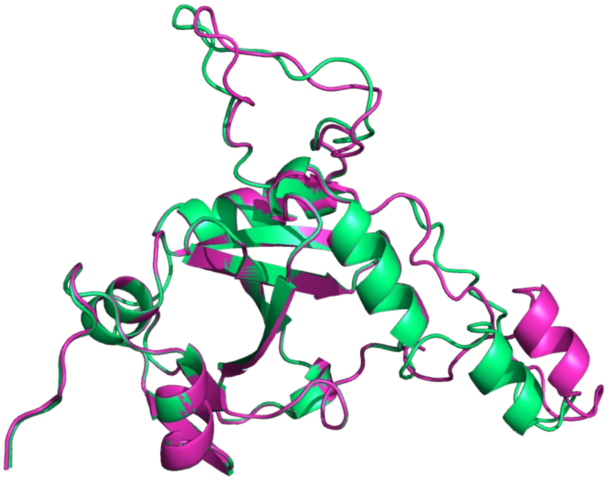

v)

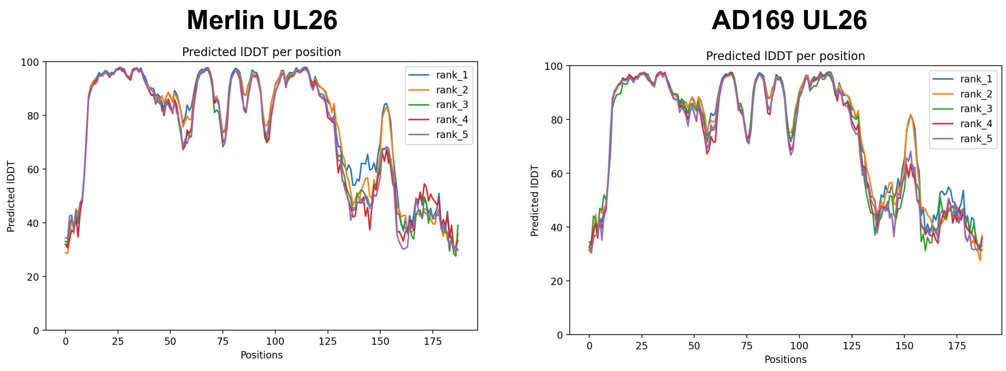
Merlin UL26
AD169 UL26
Predicted IDDT per position
Predicted IDDT
Positions
rank_1
rank_2
rank_3
rank_4
rank_5

C- UL31

i)

```
Merlin    MGDKPTLVTLLTVAVSSPPPSSPLPLVSFTELLLPPPSVAAAAVAATATSEVGEKTAEQE   60
AD169     MGDKPTLVTLLTVAVSSPPPSSPLPLVSFTELLLPPPSVAAAAVAATATSEVGEKTAEQE   60
          ************************************************************

Merlin    VAAAGPETRNERRENREDEGGETRTTGTTAVKRSHDGIPRQLAERLRLCRHMDPEQDYRL   120
AD169     VAAADPETGNERRENRENEGGETRTTGTTAVKRSHDGIPRQLAERLRLCRHMDPEQDYRL   120
          ****.*** *********:*****************************************

Merlin    PAQDVVTSWIEALRDADRDNYGRCVRHAKIHRSASHLTAYESYLVSITEQYNTASNVTEK   180
AD169     PAQDVVTSWIEALRDADRDNYGRCVRHAKIHRSASHLTAYESYLVSITEQYNTASNVTEK   180
          ************************************************************

Merlin    ASYVQGCIFLSFPVIYNNTQGCGYKYDWSNVVTPKAAYAELFFLLCSTSESSVVLQPLIT   240
AD169     ASYVQGCIFLSFPVIYNNTQGCGYKYDWSNVVTPKAAYAELFFLLCSTSESSVVLQPLIT   240
          ************************************************************

Merlin    KGGLCSSMAVYDEETMRQSQAVQIGFLHTQLVMVPFVPHACPHYAVPFTTPGKPGCGGAP   300
AD169     KGGLCSSMAVYDEETMRQSQAVQIGFLHTQLVMVPFVPHACPHYAVPFTTPGKPGCGGAP   300
          ************************************************************

Merlin    SGVAGLEETAPFGRVSVTRHGATLLCRVDHLTWISKRVTTYGHKKITRYLAQFRGTMDDD   360
AD169     SGVAGLEEAAPFGRVSVTRHGATLLCRVDHLTWISKRVTTYGHKKITRYLAQFRGTMDDD   360
          ********:***************************************************

Merlin    EAALPGEDEAWIASKNVQYEFMGLIFTVNVDSLCVDAEQRQLLGTVATSFCHRVSDKITA   420
AD169     EAALPGEDEAWIASKNVQYEFMGLIFTVNVDSLCVDAEQRQLLGTVATSFCHRVSDKITA   420
          ************************************************************

Merlin    RNMPRAFSFYLLTSAQRGYDLRFSRNPSLFFSGDALNCPLLNEPNVFSLTVHAPYDIHFG   480
AD169     RNMPRAFSFYLLTSAQRGYDLRFSRNPSLFFSGDALNCPLLNEPNVFSLTVHAPYDIHFG   480
          ************************************************************

Merlin    VQPRQTVELDLRYVQITDRCFLVANLPHEDAFYTGLSVWRGGEPLKVTLWTRTRSIVIPQ   540
AD169     VQPRQTVELDLRYVQITDRCFLVANLPHEDAFYTGLSVWRGGEPLKVTLWTRTRSIVIPQ   540
          ************************************************************

Merlin    GTPIATLYQITEGDGNVYSYNHHTVFRQMHAAGATTFFLGDMQLPADNFLTSPHP   595
AD169     GTPIATLYQITEGDGNVYSYNHHTVFRQMHAAGTTTFFLGDMQLPADNFLTSPHP   595
          *********************************:*********************
```

ii)

```
Merlin          MGDKPTLVTLLTVAVSSPPPSSPLPLVSFTELLLPPPSVAAAAVAATATSEVGEKTAEQE    60
TB40/E          MGDKPTLVTLLTVAVSSPPPSSPLPLVSFTELLLPPPSVAAAAVAATATSEVGEKTAEQE    60
JP              MGDKPTLVTLLTVAVSSPPPSSPLPLVSFTELLLPPPSVAAAAVAATATSEVGEKTAERE    60
JHC             MGDKPTLVTLLTVAVSSPPPSSPLPLVSFTELLLPPPSVAAAAVAATATSEVGEKTAEQE    60
HAN1            MGDKPTLVTLLTVAVSSPPPSSPLPLVSFTELLLPPPSVAAAAVAATATSEVGEKTAEQE    60
AD169           MGDKPTLVTLLTVAVSSPPPSSPLPLVSFTELLLPPPSVAAAAVAATATSEVGEKTAEQE    60
Toledo          MGDKPALVTLLTVAVSSPPPSSPLPLVSFTELLLPPPSVAAAAVAATATSEVGEKTAEQE    60
Davis           MGDKPTLVTLLTVAVSSPPPSSPLPLVSFTELLLPPPSVAAAAVAATATSEVGEKTAEQE    60
Towne           MGDKPTLVTLLTVAVSSPPPSSPLPLVSFTELLLPPPSVAAAAVAATATSEVGEKTAEQE    60
TR              MGEKPTLVTLLTVAVSSPPPSSPLPLVSFTELLLPPPSVAAAAVAATATSEVGEKTAEQE    60
                **:**:****************************************************:*

Merlin          VAAAGPETRNERRENREDEGGETRTTGTTAVKRSHDGIPRQLAERLRLCRHMDPEQDYRL    120
TB40/E          VAAAGPETGNERRENREDEGGETRTTGTTAVKRSHDGIPRQLAERLRLCRHMDPEQDYRL    120
JP              VAAAGPETGNERRENREDERGETRTTGTTAVKRSHDGIPRQLAERLRLCRHMDPEQDYRL    120
JHC             VAAAGPETGNERRENRENEGGETRTTGTTAVKRSHDGIPRQLAERLRLCRHMDPEQDYRL    120
HAN1            VAAAGPETGNERRENREDEGGETRTTGTTAVKRSHDGIPRQLAERLRLCRHMDPEQDYRL    120
AD169           VAAADPETGNERRENRENEGGETRTTGTTAVKRSHDGIPRQLAERLRLCRHMDPEQDYRL    120
Toledo          VAAAGPETGNERRENRENEGGETRTTGTTAVKRSHDGIPRQLAERLRLCRHMDPEQDYRL    120
Davis           VAAAGPETGNERRENREDEGGETRTTGTTAVKRSHDGIPRQLAERLRLCRHMDPEQDYRL    120
Towne           VAAAGPETGNERRENREDEGGETRTTGTTAVKRSHDGIPRQLAERLRLCRHMDPEQDYRL    120
TR              VAAAGPETGNERRENRENEGGETRTTDTTAVKRSHDGIPRQLAERLRLCRHMDPEQDYRL    120
                ****.*** ********:* ******.*********************************

Merlin          PAQDVVTSWIEALRDADRDNYGRCVRHAKIHRSASHLTAYESYLVSITEQYNTASNVTEK    180
TB40/E          PAQDVVTSWIEALRDADRDNYGRCVRHAKIHRSASHLTAYESYLVSITEQYNTASNVTEK    180
JP              PAQDVVTSWIEALRDADRDNYGRCVRHAKIHRSASHLTAYESYLVSITEQYNTASNVTEK    180
JHC             PAQDVVTSWIEALRDADRDNYGRCVRHAKIHRSASHLTAYESYLVSITEQYNTASNVTEK    180
HAN1            PAQDVVTSWIEALRDADRDNYGRCVRHAKIHRSASHLTAYESYLVSITEQYNTASNVTEK    180
AD169           PAQDVVTSWIEALRDADRDNYGRCVRHAKIHRSASHLTAYESYLVSITEQYNTASNVTEK    180
Toledo          PAQDVVTSWIEALRDADRDNYGRCVRHAKIHRSASHLTAYESYLVSITEQYNTASNVTEK    180
Davis           PAQDVVTSWIEALRDADRDNYGRCVRHAKIHRSASHLTAYESYLVSITEQYNTASNVTEK    180
Towne           PAQDVVTSWIEALRDADRDNYGRCVRHAKIHRSASHLTAYESYLVSITEQYNTASNVTEK    180
TR              PAQDVVTSWIEALRDADRDNYGRCVRHAKIHRSASHLTAYESYLVSITEQYNTASNVTEK    180
                ************************************************************

Merlin          ASYVQGCIFLSFPVIYNNTQGCGYKYDWSNVVTPKAAYAELFFLLCSTSESSVVLQPLIT    240
TB40/E          ASYVQGCIFLSFPVIYNNTQGCGYKYDWSNVVTPKAAYAELFFLLCSTSESSVVLQPLIT    240
JP              ASYVQGCIFLSFPVIYNNTQGCGYKYDWSNVVTPKAAYAELFFLLCSTSESSVVLQPLIT    240
JHC             ASYVQGCIFLSFPVIYNNTQGCGYKYDWSNVVTPKAAYAELFFLLCSTSESSVVLQPLIT    240
HAN1            ASYVQGCIFLSFPVIYNNTQGCGYKYDWSNVVTPKAAYAELFFLLCSTSESSVVLQPLIT    240
AD169           ASYVQGCIFLSFPVIYNNTQGCGYKYDWSNVVTPKAAYAELFFLLCSTSESSVVLQPLIT    240
Toledo          ASYVQGCIFLSFPVIYNNTQGCGYKYDWSNVVTPKAAYAELFFLLCSTSESSVVLQPLIT    240
Davis           ASYVQGCIFLSFPVIYNNTQGCGYKYDWSNVVTPKAAYAELFFLLCSTSESSVVLQPLIT    240
Towne           ASYVQGCIFLSFPVIYNNTQGCGYKYDWSNVVTPKAAYAELFFLLCSTSESSVVLQPLIT    240
TR              ASYVQGCIFLSFPVIYNNTQGCGYKYDWSNVVTPKAAYAELFFLLCSTSESSVVLQPLIT    240
                ************************************************************

Merlin          KGGLCSSMAVYDEETMRQSQAVQIGFLHTQLVMVPFVPHACPHYAVPFTTPGKPGCGGAP    300
TB40/E          KGGLCSSMAVYDEETMRQSQAVQIGFLHTQLVMVPFVPHACPHYAVPFTTPGKPGCGGAP    300
JP              KGGLCSSMAVYDEETMRQSQAVQIGFLHTQLVMVPFVPHACPHYAVPFTTPGKPGCGGAP    300
JHC             KGGLCSSMAVYDEETMRQSQAVQIGFLHTQLVMVPFVPHACPHYAVPFTTPGKPGCGGAP    300
HAN1            KGGLCSSMAVYDEETMRQSQAVQIGFLHTQLVMVPFVPHACPHYAVPFTTPGKPGCGGAP    300
AD169           KGGLCSSMAVYDEETMRQSQAVQIGFLHTQLVMVPFVPHACPHYAVPFTTPGKPGCGGAP    300
Toledo          KGGLCSSMAVYDEETMRQSQAVQIGFLHTQLVMVPFVPHACPHYAVPFTTPGKPGCGGAP    300
Davis           KGGLCSSMAVYDEETMRQSQAMQIGFLHTQLVMVPFVPHACPHYAVPFTTPGKPGCGGAP    300
Towne           KGGLCSSMAVYDEETMRQSQAVQIGFLHTQLVMVPFVPHACPHYAVPFTTPGKPGCGGAP    300
TR              KGGLCSSMAVYDEETMRQSQAVQIGFLHTQLVMVPFVPHACPHYAVPFTTPGKPGCGGAP    300
                *********************:**************************************
```

```
Merlin      SGVAGLEETAPFGRVSVTRHGATLLCRVDHLTWISKRVTTYGHKKITRYLAQFRGTMDDD    360
TB40/E      SGVAGLEEAAPFGRVSVTRHGATLLCRVDHLTWISKRVTTYGHKKITRYLAQFRGTMDDD    360
JP          SGVAGLEEAAPFGRVSVTRHGATLLCRVDHLTWISKRVTTYGHKKITRYLAQFRGTMDDD    360
JHC         SGVAGLEEAAPFGRVSVTRHGATLLCRVDHLTWISKRVTTYGHKKITRYLAQFRGTMDDD    360
HAN1        SGVAGLEETAPFGRVSVTRHGATLLCRVDHLTWISKRVTTYGHKKITRYLAQFRGTMDDD    360
AD169       SGVAGLEEAAPFGRVSVTRHGATLLCRVDHLTWISKRVTTYGHKKITRYLAQFRGTMDDD    360
Toledo      SGVAGLEEAAPFGRVSVTRHGATLLCRVDHLTWISKRVTTYGHKKITRYLAQFRGTMDDD    360
Davis       SGVAGLEETAPFGRVSVTRHGATLLCRVDHLTWISKRVTTYGHKKITRYLAQFRGTMDDD    360
Towne       SGVAGLEEAAPFGRVSVTRHGATLLCRVDHLTWISKRVTTYGHKKITRYLAQFRGTMDDD    360
TR          SGVAGLEEAAPFGRVSVTRHGATLLCRVDHLTWISKRVTTYGHKKITRYLAQFRGTMDDD    360
            ********:***************************************************

Merlin      EAALPGEDEAWIASKNVQYEFMGLIFTVNVDSLCVDAEQRQLLGTVATSFCHRVSDKITA    420
TB40/E      EAALPGEDEAWIASKNVQYEFMGLIFTVNVDSLCVDAEQRQLLGTVATSFCHRVSDKITA    420
JP          EAALPGEDEAWIASKNVQYEFMGLIFTVNVDSLCVDAEQRQLLGTVATSFCHRVSDKITA    420
JHC         EAALPGEDEAWIASKNVQYEFMGLIFTVNVDSLCVDAEQRQLLGTVATSFCHRVSDKITA    420
HAN1        EAALPGEDEAWIASKNVQYEFMGLIFTVNVDSLCVDAEQRQLLGTVATSFCHRVSDKITA    420
AD169       EAALPGEDEAWIASKNVQYEFMGLIFTVNVDSLCVDAEQRQLLGTVATSFCHRVSDKITA    420
Toledo      EAALPGEDEAWIASKNVQYEFMGLIFTVNVDSLCVDAEQRQLLGTVATSFCHRVSDKITA    420
Davis       EAALPGEDEAWIASKNVQYEFMGLIFTVNVDSLCVDAEQRQLLGTVATSFCHRVSDKITA    420
Towne       EAALPGEDEAWIASKNVQYEFMGLIFTVNVDSLCVDAEQRQLLGTVATSFCHRVSDKITA    420
TR          EAALPGEDEAWIASKNVQYEFMGLIFTVNVDSLCVDAEQRQLLGTVATSFCHRVSDKITA    420
            ************************************************************

Merlin      RNMPRAFSFYLLTSAQRGYDLRFSRNPSLFFSGDALNCPLLNEPNVFSLTVHAPYDIHFG    480
TB40/E      RNMPRAFSFYLLTSAQRGYDLRFSRNPSLFFSGDALNCPLLNEPNVFSLTVHAPYDIHFG    480
JP          RNMPRAFSFYLLTSAQRGYDLRFSRNPSLFFSGDALNCPLLNEPNVFSLTVHAPYDIHFG    480
JHC         RNMPRAFSFYLLTSAQRGYDLRFSRNPSLFFSGDALNCPLLNEPNVFSLTVHAPYDIHFG    480
HAN1        RNMPRAFSFYLLTSAQRGYDLRFSRNPSLFFSGDALNCPLLNEPNVFSLTVHAPYDIHFG    480
AD169       RNMPRAFSFYLLTSAQRGYDLRFSRNPSLFFSGDALNCPLLNEPNVFSLTVHAPYDIHFG    480
Toledo      RNMPRAFSFYLLTSAQRGYDLRFSRNPSLFFSGDALNCPLLNEPNVFSLTVHAPYDIHFG    480
Davis       RNMPRAFSFYLLTSPQRGYDLRFSRNPSLFFSGDALNCPLLNEPNVFSLTVHAPYDIHFG    480
Towne       RNMPRAFSFYLLTSAQRGYDLRFSRNPSLFFSGDALNCPLLNEPNVFSLTVHAPYDIHFG    480
TR          RNMPRAFSFYLLTSAQRGYDLRFSRNPSLFFSGDALNCPLLNEPNVFSLTVHAPYDIHFG    480
            ************** *********************************************

Merlin      VQPRQTVELDLRYVQITDRCFLVANLPHEDAFYTGLSVWRGGEPLKVTLWTRTRSIVIPQ    540
TB40/E      VQPRQTVELDLRYVQITDRCFLVANLPHEDAFYTGLSVWRGGEPLKVTLWTRTRSIVIPQ    540
JP          VQPRQTVELDLRYVQITDRCFLVANLPHEDAFYTGLSVWRGGEPLKVTLWTRTRSIVIPQ    540
JHC         VQPRQTVELDLRYVQITDRCFLVANLPHEDAFYTGLSVWRGGEPLKVTLWTRTRSIVIPQ    540
HAN1        VQPRQTVELDLRYVQITDRCFLVANLPHEDAFYTGLSVWRGGEPLKVTLWTRTRSIVIPQ    540
AD169       VQPRQTVELDLRYVQITDRCFLVANLPHEDAFYTGLSVWRGGEPLKVTLWTRTRSIVIPQ    540
Toledo      VQPRQTVELDLRYVQITDRCFLVANLPHEDAFYTGLSVWRGGEPLKVTLWTRTRSIVIPQ    540
Davis       VQPRQTVELDLRYVQITDRCFLVANLPHEDAFYTGLSVWRGGEPLKVTLWTRTRSIVIPQ    540
Towne       VQPRQTVELDLRYVQITDRCFLVANLPHEDAFYTGLSVWRGGEPLKVTLWTRTRSIVIPQ    540
TR          VQPRQTVELDLRYVQITDRCFLVANLPHEDAFYTGLSVWRGGEPLKVTLWTRTRSIVIPQ    540
            ************************************************************

Merlin      GTPIATLYQITEGDGNVYSYNHHTVFRQMHAAGATTFFLGDMQLPADNFLTSPHP         595
TB40/E      GTPIATLYQITEGDGNVYSYNHHTVFRQMHAAGATTFFLGDMQLPADNFLTSPHP         595
JP          GTPIATLYQITEGDGNVYSYNHHTVFRQMHAAGATTFFLGDMQLPADNFLTSPHP         595
JHC         GTPIATLYQITEGDGNVYSYNHHTVFRQMHAAGATTFFLGDMQLPADNFLTSPHP         595
HAN1        GTPIATLYQITEGDGNVYSYNHHTVFRQMHAAGATTFFLGDMQLPADNFLTSPHP         595
AD169       GTPIATLYQITEGDGNVYSYNHHTVFRQMHAAGTTTFFLGDMQLPADNFLTSPHP         595
Toledo      GTPIATLYQITEGDGNVYSYNHHTVFRQMHAAGATTFFLGDMQLPADNFLTSPHP         595
Davis       GTPIATLYQITEGDGNVYSYNHHTVFRQMHAAGTTTFFLGDMQLPADNFLTSPHP         595
Towne       GTPIATLYQITEGDGNVYSYNHHTVFRQMHAAGATTFFLGDMQLPADNFLTSPHP         595
TR          GTPIATLYQITEGDGNVYSYNHHTVFRQMHAAGATTFFLGDMQLPADNFLTSPHP         595
            *********************************:*********************
```

iii)

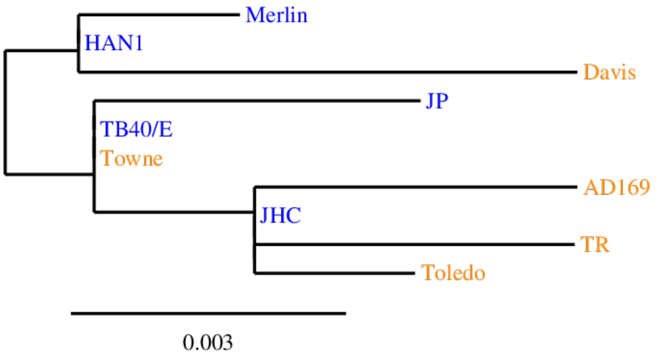
Merlin
HAN1
Davis
JP
TB40/E
Towne
AD169
JHC
TR
Toledo
0.003

iv)

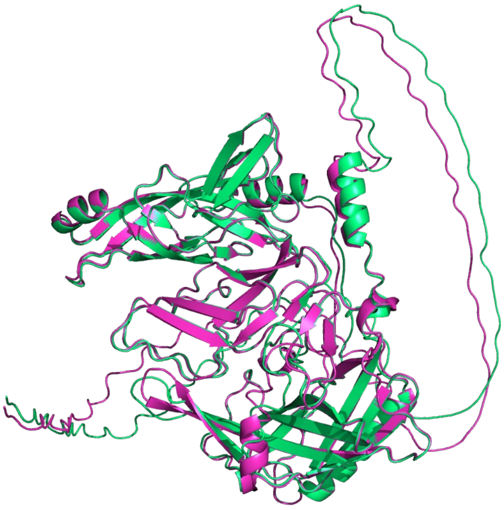

v)

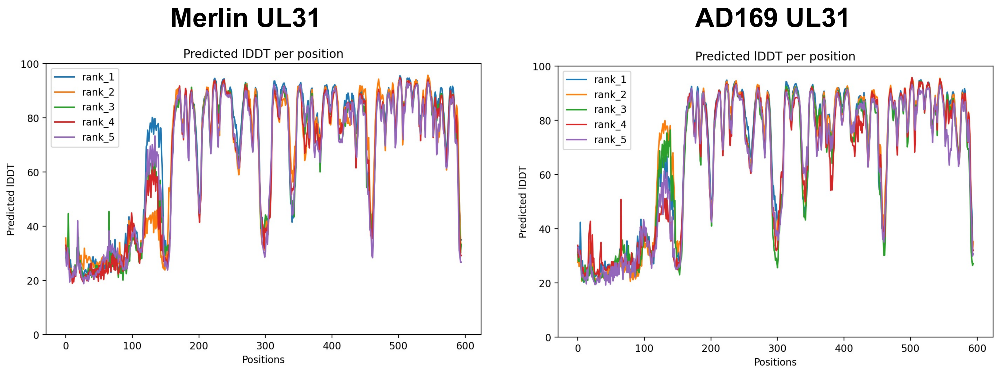
Merlin UL31
Predicted lDDT per position
Predicted lDDT
Positions
rank_1
rank_2
rank_3
rank_4
rank_5
AD169 UL31
Predicted lDDT per position
Predicted lDDT
Positions
rank_1
rank_2
rank_3
rank_4
rank_5

D- UL82

i)

```
Merlin    MSQASSSPGEGPSSEAAAISEAEAASGSFGRLHCQVLRLITNVEGGSLEAGRLRLLDLRT   60
AD169     MSQASSSPGEGPSSEAAAISEAEAASGSFGRLHCQVLRLITNVEGGSLEAGRLRLLDLRT   60
          ************************************************************

Merlin    NIEVSRPSVLCCFQENKSPHDTVDLTDLNIKGRCVVGEQDRLLVDLNNFGPRRLTPGSEN   120
AD169     NIEVSRPSVLCCFQENKSPHDTVDLTDLNIKGRCVVGEQDRLLVDLNNFGPRRLTPGSEN   120
          ************************************************************

Merlin    NTVSVLAFALPLDRVPVSGLHLFQSQRRGGEENRPRMEARAIIRRTAHHWAVRLTVTPNW   180
AD169     NTVSVLAFALPLDRVPVSGLHLFQSQRRGGEENRPRMEARAIIRRTAHHWAVRLTVTPNW   180
          ************************************************************

Merlin    RRRTDSSLEAGQIFVSQFAFRAGAIPLTLVDALEQLACSDPNTYIHKTETDERGQWIMLF   240
AD169     RRRTDSSLEAGQIFVSQFAFRAGAIPLTLVDALEQLACSDPNTYIHKTETDERGQWIMLF   240
          ************************************************************

Merlin    LHHDSPHPPTSVFLHFSVYTHRAEVVARHNPYPHLRRLPDNGFQLLIPKSFTLTRIHPEY   300
AD169     LHHDSPHPPTSVFLHFSVYTHRAEVVARHNPYPHLRRLPDNGFQLLIPKSFTLTRIHPEY   300
          ************************************************************

Merlin    IVQIQNAFETNQTHDTIFFPENIPGVSIEAGPLPDRVRITLRVTLTGDQAVHLEHRQPLG   360
AD169     IVQIQNAFETNQTHDTIFFPENIPGVSIEAGPLPDRVRITLRVTLTGDQAVHLEHRQPLG   360
          ************************************************************

Merlin    RIHFFRRGFWTLTPGKPDKIKRPQVQLRAGLFPRSNVMRGAVSEFLPQSPGLPPTEEEEE   420
AD169     RIHFFRRGFWTLTPGKPDKIKRPQVQLRAGLFPRSNVMRGAVSEFLPQSPGLPPTEEEEE   420
          ************************************************************

Merlin    EEEEDDEDDLSSTPTPTPLSEAMFAGFEEASGDEDSDTQAGLSRALILTGQRRRSGNNGA   480
AD169     EEEEDDEDDLSSTPTPTPLSEAMFAGFEEASGDEDSDTQAGLSPALILTGQRRRSGNNGA   480
          ******************************************* ****************

Merlin    LTLVIPSWHVFASLDDLVPLTVSVQHAALRPTSYLRSDMDGDVRTAADISSTLRSVPAPR   540
AD169     LTLVIPSWHVFASLDDLVPLTVSVQHAALRPTSYLRSDMDGDVRTAADISSTLRSVPAPR   540
          ************************************************************

Merlin    PSPISTASTSSTPRSRPRI                                            559
AD169     PSPISTASTSSTPRSRPRI                                            559
          *******************
```

ii)

```
Merlin      MSQASSSPGEGPSSEAAAISEAEAASGSFGRLHCQVLRLITNVEGGSLEAGRLRLLDLRT   60
TB40/E      MSQASSSPGEGPSSEAAAISEAEAASGSFGRLHCQVLRLITNVEGGSLEAGRLRLLDLRT   60
JP          MSQASSSPGEGPSSEAAAISEAEAASGSFGRLHCQVLRLITNVEGGSLEAGRLRLLDLRT   60
JHC         MSQASSSPGEGPSSEAAAISEAEAASGSFGRLHCQVLRLITNVEGGSLEAGRLRLLDLRT   60
HAN1        MSQASSSPGEGPSSEAAAISEAEAASGSFGRLHCQVLRLITNVEGGSLEAGRLRLLDLRT   60
AD169       MSQASSSPGEGPSSEAAAISEAEAASGSFGRLHCQVLRLITNVEGGSLEAGRLRLLDLRT   60
Toledo      MSQASSSPGEGPSSEAAAISEAEAASGSFGRLHCQVLRLITNVEGGSLEAGRLRLLDLRT   60
Davis       MSQASSSPGEGPSSEAAAISEAEAASGSFGRLHCQVLRLITNVEGGSLEAGRLRLLDLRT   60
Towne       MSQASSSPGEGPSSEAAAISEAEAASGSFGRLHCQVLRLITNVEGGSLEAGRLRLLDLRT   60
TR          MSQASSSPGEGPSSEAAAISEAEAASGSFGRLHCQVLRLITNVEGGSLEAGRLRLLDLRT   60
            ************************************************************

Merlin      NIEVSRPSVLCCFQENKSPHDTVDLTDLNIKGRCVVGEQDRLLVDLNNFGPRRLTPGSEN   120
TB40/E      NIEVSRPSVLCCFQENKSPHDTVDLTDLNIKGRCVVGEQDRLLVDLNNFGPRRLTPGSEN   120
JP          NIEVSRPSVLCCFQENKSPHDTVDLTDLNIKGRCVVGEQDRLLVDLNNFGPRRLTPGSEN   120
JHC         NIEVSRPSVLCCFQENKSPHDTVDLTDLNIKGRCVVGEQDRLLVDLNNFGPRRLTPGSEN   120
HAN1        NIEVSRPSVLCCFQENKSPHDTVDLTDLNIKGRCVVGEQDRLLVDLNNFGPRRLTPGSEN   120
AD169       NIEVSRPSVLCCFQENKSPHDTVDLTDLNIKGRCVVGEQDRLLVDLNNFGPRRLTPGSEN   120
Toledo      NIEVSRPSVLCCFQENKSPHDTVDLTDLNIKGRCVVGEQDRLLVDLNNFGPRRLTPGSEN   120
Davis       NIEVSRPSVLCCFQENKSPHDTVDLTDLNIKGRCVVGEQDRLLVDLNNFGPRRLTPGSEN   120
Towne       NIEVSRPSVLCCFQENKSPHDTVDLTDLNIKGRCVVGEQDRLLVDLNNFGPRRLTPGSEN   120
TR          NIEVSRPSVLCCFQENKSPHDTVDLTDLNIKGRCVVGERDRLLVDLNNFGPRRLTPGSEN   120
            **************************************:*********************

Merlin      NTVSVLAFALPLDRVPVSGLHLFQSQRRGGEENRPRMEARAIIRTAHHWAVRLTVTPNW   180
TB40/E      NTVSVLAFALPLDRVPVSGLHLFQSQRRGGEENRPRVEARAIIRTAHYWAVRLTVTPNW   180
JP          NTVSVLAFALPLDRVPVSGLHLFQSQRRGGEENRPRVEARAIIRTAHYWAVRLTVTPNW   180
JHC         NTVSVLAFALPLDRVPVSGLHLFQSQRRGGEENRPRMEARAIIRTAHHWAVRLTVTPNW   180
HAN1        NTVSVLAFALPLDRVPVSGLHLFQSQRRGGEENRPRMEARAIIRTAHHWAVRLTVTPNW   180
AD169       NTVSVLAFALPLDRVPVSGLHLFQSQRRGGEENRPRMEARAIIRTAHHWAVRLTVTPNW   180
Toledo      NTVSVLAFALPLDRVPVSGLHLFQSQRRGGEENRPRVEARAIIRTAHYWAVRLTVTPNW   180
Davis       NTVSVLAFALPLDRVPVSGLHLFQSQRRGGEENRPRMEARAIIRTAHHWAVRLTVTPNW   180
Towne       NTVSVLAFALPLDRVPVSGLHLFQSQRRGGEENRPRVEARAIIRTAHYWAVRLTVTPNW   180
TR          NTVSVLAFALPLDRVPVSGLHLFQSQRRGGEENRPRMEARAIIRTAHHWAVRLTVTPNW   180
            ************************************:***********:***********

Merlin      RRRTDSSLEAGQIFVSQFAFRAGAIPLTLVDALEQLACSDPNTYIHKTETDERGQWIMLF   240
TB40/E      RRRTDSSLEAGQIFVSQFAFRAGAIPLTLVDALEQLACSDPNTYIHKTETDERGQWIMLF   240
JP          RRRTDSSLEAGQIFVSQFAFRAGAIPLTLVDALEQLACSDPNTYIHKTETDERGQWIMLF   240
JHC         RRRTDSSLEAGQIFVSQFAFRAGAIPLTLVDALEQLACSDPNTYIHKTETDERGQWIMLF   240
HAN1        RRRTDSSLEAGQIFVSQFAFRAGAIPLTLVDALEQLACSDPNTYIHKTETDERGQWIMLF   240
AD169       RRRTDSSLEAGQIFVSQFAFRAGAIPLTLVDALEQLACSDPNTYIHKTETDERGQWIMLF   240
Toledo      RRRTDSSLEAGQIFVSQFAFRAGAIPLTLVDALEQLACSDPNTYIHKTETDERGQWIMLF   240
Davis       RRRTDSSLEAGQIFVSQFAFRAGAIPLTLVDALEQLACSDPNTYIHKTETDERGQWIMLF   240
Towne       RRRTDSSLEAGQIFVSQFAFRAGAIPLTLVDALEQLACSDPNTYIHKTETDERGQWIMLF   240
TR          RRRTDSSLEAGQIFVSQFAFRAGAIPLTLVDALEQLACSDPNTYIHKTETDERGQWIMLF   240
            ************************************************************

Merlin      LHHDSPHPPTSVFLHFSVYTHRAEVVARHNPYPHLRRLPDNGFQLLIPKSFTLTRIHPEY   300
TB40/E      LHHDSPHPPTSVFLHFSVYTHRAEVVARHNPYPHLRRLPDNGFQLLIPKSFTLTRIHPEY   300
JP          LHHDSPHPPTSVFLHFSVYTHRAEVVARHNPYPHLRRLPDNGFQLLIPKSFTLTRIHPEY   300
JHC         LHHDSPHPPTSVFLHFSVYTHRAEVVARHNPYPHLRRLPDNGFQLLIPKSFTLTRIHPEY   300
HAN1        LHHDSPHPPTSVFLHFSVYTHRAEVVARHNPYPHLRRLPDNGFQLLIPKSFTLTRIHPEY   300
AD169       LHHDSPHPPTSVFLHFSVYTHRAEVVARHNPYPHLRRLPDNGFQLLIPKSFTLTRIHPEY   300
Toledo      LHHDSPHPPTSVFLHFSVYTHRAEVVARHNPYPHLRRLPDNGFQLLIPKSFTLTRIHPEY   300
Davis       LHHDSPHPPTSVFLHFSVYTHRAEVVARHNPYPHLRRLPDNGFQLLIPKSFTLTRIHPEY   300
Towne       LHHDSPHPPTSVFLHFSVYTHRAEVVARHNPYPHLRRLPDNGFQLLIPKSFTLTRIHPEY   300
TR          LHHDSPHPPTSVFLHFSVYTHRAEVVARHNPYPHLRRLPDNGFQLLIPKSFTLTRIHPEY   300
            ************************************************************
```

```
Merlin      IVQIQNAFETNQTHDTIFFPENIPGVSIEAGPLPDRVRITLRVTLTGDQAVHLEHRQPLG   360
TB40/E      IVQIQNAFETNQTHDTIFFPENIPGVSIEAGPLPDRVRITLRVTLTGDQAVHLEHRQPLG   360
JP          IVQIQNAFETNQTHDTIFFPENIPGVSIEAGPLPDRVRITLRVTLTGDQAVHLEHRQPLG   360
JHC         IVQIQNAFETNQTHDTIFFPENIPGVSIEAGPLPDRVRITLRVTLTGDQAVHLEHRQPLG   360
HAN1        IVQIQNAFETNQTHDTIFFPENIPGVSIEAGPLPDRVRITLRVTLTGDQAVHLEHRQPLG   360
AD169       IVQIQNAFETNQTHDTIFFPENIPGVSIEAGPLPDRVRITLRVTLTGDQAVHLEHRQPLG   360
Toledo      IVQIQNAFETNQTHDTIFFPENIPGVSIEAGPLPDRVRITLRVTLTGDQAVHLEHRQPLG   360
Davis       IVQIQNAFETNQTHDTIFFPENIPGVSIEAGPLPDRVRITLRVTLTGDQAVHLEYRQPLG   360
Towne       IVQIQNAFETNQTHDTIFFPENIPGVSIEAGPLPDRVRITLRVTLTGDQAVHLEHRQPLG   360
TR          IVQIQNAFETNQTHDTIFFPENIPGVSIEAGPLPDRVRITLRVTLTGDQAVHLEHRQPLG   360
            ******************************************************:*****

Merlin      RIHFFRRGFWTLTPGKPDKIKRPQVQLRAGLFPRSNVMRGAVSEFLPQSPGLPPTEEEEE   420
TB40/E      RIHFFRRGFWTLTPGKPDKIKRPQVQLRAGLFPRSNVMRGAVSEFLPQSPGLPPTEEEEE   420
JP          RIHFFRRGFWTLTPGKPDKIKRPQVQLRAGLFPRSNVMRGAVSEFLPQSPGLPPTEEEEE   420
JHC         RIHFFRRGFWTLTPGKPDKIKRPQVQLRAGLFPRSNVMRGAVSEFLPQSPGLPPTEEEEE   420
HAN1        RIHFFRRGFWTLTPGKPDKIKRPQVQLRAGLFPRSNVMRGAVSEFLPQSPGLPPTEEEEE   420
AD169       RIHFFRRGFWTLTPGKPDKIKRPQVQLRAGLFPRSNVMRGAVSEFLPQSPGLPPTEEEEE   420
Toledo      RIHFFRRGFWTLTPGKPDKIKRPQVQLRAGLFPRSNVMRGAVSEFLPQSPGLPPTEEEEE   420
Davis       RIHFFRRGFWTLTPGKPDKIKRPQVQLRAGLFPRSNVMRGAVSEFLPQSPGLPPTEEEEE   420
Towne       RIHFFRRGFWTLTPGKPDKIKRPQVQLRAGLFPRSNVMRGAVSEFLPQSPGLPPTEEEEE   420
TR          RIHFFRRGFWTLTPGKPDKIKRPQVQLRAGLFPRSDVVRGAVSEFLPQSPGLPPTEEEEE   420
            ***********************************:*:**********************

Merlin      E-EEEDDEDDLSSTPTPTPLSEAMFAGFEEASGDEDSDTQAGLSRALILTGQRRRSGNNG   479
TB40/E      E-EEEDDEDDLSSTPTPTPLSEAMFAGFEEASGDEDSDTQAGLSRALILTGQRRRSGNNG   479
JP          E-EEEDDEDDLSSTPTPTPLSEAMFAGFEEASGDEDSDTQAGLSRALILTGQRRRSGNNG   479
JHC         E-EEEDDEDDLSSTPTPTPLSEAMFAGFEEASGDEDSDTQAGLSRALILTGQRRRSGNNG   479
HAN1        E-EEEDDEDDLSSTPTPTPLSEAMFAGFEEASGDEDSDTQAGLSRALILTGQRRRSGNNG   479
AD169       E-EEEDDEDDLSSTPTPTPLSEAMFAGFEEASGDEDSDTQAGLSPALILTGQRRRSGNNG   479
Toledo      EEEEEDDDDDLSSTPTPTPLSEAMFAGFEEASGDEDSDTQAGLSRALILTGQRRRSGNNG   480
Davis       E-EEEDDEDDLSSTPTPTPLSEAMFAGFEEASGDEDSDTQAGLSRALILTGQRRRSGNNG   479
Towne       E-EEEDDEDDLSSTPTPTPLSEAMFAGFEEASGDEDSDTQAGLSRALILTGQRRRSGNNG   479
TR          E-EEEDDEDDLSSTPTPTPLSEAMFAGFEEASGDEDSDTQAGLSRALILTGQRRRSGNNG   479
            * *****:************************************ ***************

Merlin      ALTLVIPSWHVFASLDDLVPLTVSVQHAALRPTSYLRSDMDGDVRTAADISSTLRSVPAP   539
TB40/E      ALTLVIPSWHVFASLDDLVPLTVSVQHAALRPTSYLRSDMDGDVRTAADISSTLRSVPAP   539
JP          ALTLVIPSWHVFASLDDLVPLTVSVQHAALRPTSYLRSDMDGDVRTAADISSTLRSVPAP   539
JHC         ALTLVIPSWHVFASLDDLVPLTVSVQHAALRPTSYLRSDMDGDVRTAADISSTLRSVPAP   539
HAN1        ALTLVIPSWHVFASLDDLVPLTVSVQHAALRPTSYLRSDMDGDVRTAADISSTLRSVPAP   539
AD169       ALTLVIPSWHVFASLDDLVPLTVSVQHAALRPTSYLRSDMDGDVRTAADISSTLRSVPAP   539
Toledo      ALTLVIPSWHVFASLDDLVPLTVSVQHGALRPTSYLRSDMDGDVRTAADISSTLRSVPAP   540
Davis       ALTLVIPSWHVFASLDDLVPLTVSVQHAALRPTSYLRSDMDGDVRTAADISSTLRSVPAP   539
Towne       ALTLVIPSWHVFASLDDLVPLTVSVQHAALRPTSYLRSDMDGDVRTAADISSTLRSVPAP   539
TR          ALTLVIPSWHVFASLDDLVPLTVSVQHAALRPTSYLRSDMDGDVRTAADISSTLRSVPAP   539
            ***************************.********************************

Merlin      RPSPISTASTSSTPRSRPRI                                           559
TB40/E      RPSPISTASTSSTPRSRPRI                                           559
JP          RPSPISTASTSSTPRSRPRI                                           559
JHC         RPSPISTASTSSTPRSRPRI                                           559
HAN1        RPSPISTASTSSTPRSRPRI                                           559
AD169       RPSPISTASTSSTPRSRPRI                                           559
Toledo      RPSPISTASTSSTPRSRPRI                                           560
Davis       RPSPISTASTSSTPRSRPRI                                           559
Towne       RPSPISTASTSSTPRSRPRI                                           559
TR          RPSPISTASTSSTPRSRPRI                                           559
            ********************
```

iii)

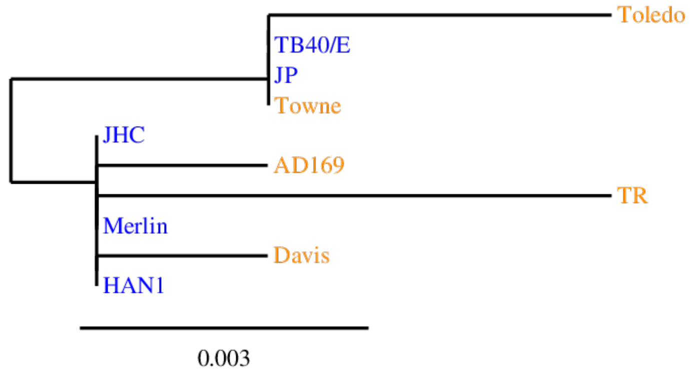
Toledo
TB40/E
JP
Towne
JHC
AD169
TR
Merlin
Davis
HAN1
0.003

iv)

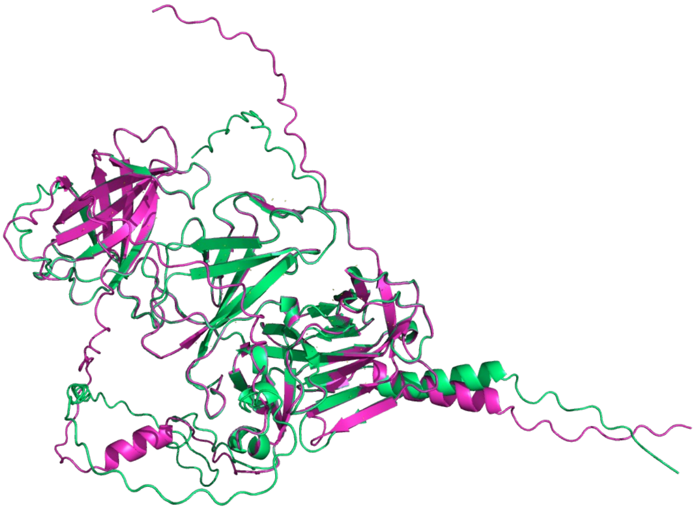

v)

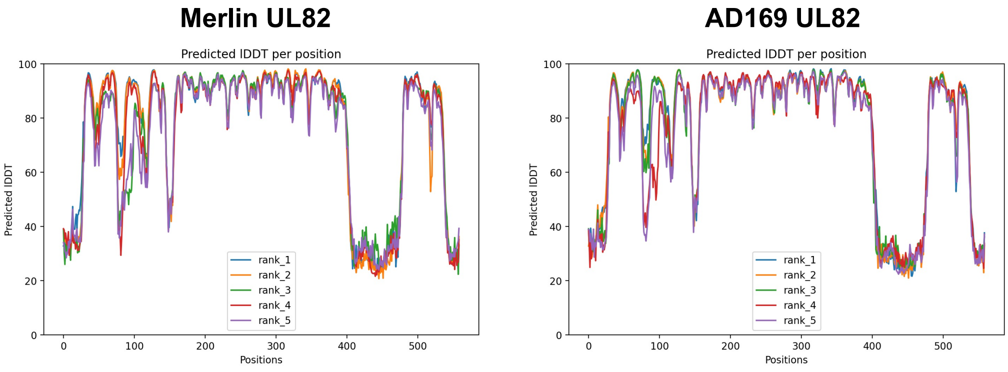
Merlin UL82
Predicted lDDT per position
Predicted lDDT
Positions
rank_1
rank_2
rank_3
rank_4
rank_5
AD169 UL82
Predicted lDDT per position
Predicted lDDT
Positions
rank_1
rank_2
rank_3
rank_4
rank_5

E- UL83

i)

```
Merlin    MESRGRRCPEMISVLGPISGHVLKAVFSRGDTPVLPHETRLLQTGIHVRVSQPSLILVSQ   60
AD169     MESRGRRCPEMISVLGPISGHVLKAVFSRGDTPVLPHETRLLQTGIHVRVSQPSLILVSQ   60
          ************************************************************

Merlin    YTPDSTPCHRGDNQLQVQHTYFTGSEVENVSVNVHNPTGRSICPSQEPMSIYVYALPLKM   120
AD169     YTPDSTPCHRGDNQLQVQHTYFTGSEVENVSVNVHNPTGRSICPSQEPMSIYVYALPLKM   120
          ************************************************************

Merlin    LNIPSINVHHYPSAAERKHRHLPVADAVIHASGKQMWQARLTVSGLAWTRQQNQWKEPDV   180
AD169     LNIPSINVHHYPSAAERKHRHLPVADAVIHASGKQMWQARLTVSGLAWTRQQNQWKEPDV   180
          ************************************************************

Merlin    YYTSAFVFPTKDVALRHVVCAHELVCSMENTRATKMQVIGDQYVKVYLESFCEDVPSGKL   240
AD169     YYTSAFVFPTKDVALRHVVCAHELVCSMENTRATKMQVIGDQYVKVYLESFCEDVPSGKL   240
          ************************************************************

Merlin    FMHVTLGSDVEEDLTMTRNPQPFMRPHERNGFTVLCPKNMIIKPGKISHIMLDVAFTSHE   300
AD169     FMHVTLGSDVEEDLTMTRNPQPFMRPHERNGFTVLCPKNMIIKPGKISHIMLDVAFTSHE   300
          ************************************************************

Merlin    HFGLLCPKSIPGLSISGNLLMNGQQIFLEVQAIRETVELRQYDPVAALFFFDIDLLLQRG   360
AD169     HFGLLCPKSIPGLSISGNLLMNGQQIFLEVQAIRETVELRQYDPVAALFFFDIDLLLQRG   360
          ************************************************************

Merlin    PQYSEHPTFTSQYRIQGKLEYRHTWDRHDEGAAQGDDDVWTSGSDSDEELVTTERKTPRV   420
AD169     PQYSEHPTFTSQYRIQGKLEYRHTWDRHDEGAAQGDDDVWTSGSDSDEELVTTERKTPRV   420
          ************************************************************

Merlin    TGGGAMASASTSAGRKRKSASSATACTAGVMTRGRLKAESTVAPEEDTDEDSDNEIHNPA   480
AD169     TGGGAMASASTSAGRKRKSASSATACTAGVMTRGRLKAESTVAPEEDTDEDSDNEIHNPA   480
          ************************************************************

Merlin    VFTWPPWQAGILARNLVPMVATVQGQNLKYQEFFWDANDIYRIFAELEGVWQPAAQPKRR   540
AD169     VFTWPPWQAGILARNLVPMVATVQGQNLKYQEFFWDANDIYRIFAELEGVWQPAAQPKRR   540
          ************************************************************

Merlin    RHRQDALPGPCIASTPKKHRG                                          561
AD169     RHRQDALPGPCIASTPKKHRG                                          561
          *********************
```

ii)

```
Merlin      MESRGRRCPEMISVLGPISGHVLKAVFSRGDTPVLPHETRLLQTGIHVRVSQPSLILVSQ   60
TB40/E      MESRGRRCPEMISVLGPISGHVLKAVFSRGDTPVLPHETRLLQTGIHVRVSQPSLILVSQ   60
JP          MESRGRRCPEMISVLGPISGHVLKAVFSRGDTPVLPHETRLLQTGIHVRVSQPSLILVSQ   60
JHC         MESRGRRCPEMISVLGPISGHVLKAVFSRGDTPVLPHETRLLQTGIHVRVSQPSLILVSQ   60
HAN1        MESRGRRCPEMISVLGPISGHVLKAVFSRGDTPVLPHETRLLQTGIHVRVSQPSLILVSQ   60
AD169       MESRGRRCPEMISVLGPISGHVLKAVFSRGDTPVLPHETRLLQTGIHVRVSQPSLILVSQ   60
Toledo      MESRGRRCPEMISVLGPISGHVLKAVFSRGDTPVLPHETRLLQTGIHVRVSQPSLILVSQ   60
Davis       MESRGRRCPEMISVLGPISGHVLKAVFSRGDTPVLPHETRLLQTGIHVRVSQPSLILVSQ   60
Towne       MESRGRRCPEMISVLGPISGHVLKAVFSRGDTPVLPHETRLLQTGIHVRVSQPSLILVSQ   60
TR          MESRGRRCPEMISVLGPISGHVLKAVFSRGDTPVLPHETRLLQTGIHVRVSQPSLILVSQ   60
            ************************************************************

Merlin      YTPDSTPCHRGDNQLQVQHTYFTGSEVENVSVNVHNPTGRSICPSQEPMSIYVYALPLKM   120
TB40/E      YTPDSTPCHRGDNQLQVQHTYFTGSEVENVSVNVHNPTGRSICPSQEPMSIYVYALPLKM   120
JP          YTPDSTPCHRGDNQLQVQHTYFTGSEVENVSVNVHNPTGRSICPSQEPMSIYVYALPLKM   120
JHC         YTPDSTPCHRGDNQLQVQHTYFTGSEVENVSVNVHNPTGRSICPSQEPMSIYVYALPLKM   120
HAN1        YTPDSTPCHRGDNQLQVQHTYFTGSEVENVSVNVHNPTGRSICPSQEPMSIYVYALPLKM   120
AD169       YTPDSTPCHRGDNQLQVQHTYFTGSEVENVSVNVHNPTGRSICPSQEPMSIYVYALPLKM   120
Toledo      YTPDSTPCHRGDNQLQVQHTYFTGSEVENVSVNVHNPTGRSICPSQEPMSIYVYALPLKM   120
Davis       YTPDSTPCHRGDNQLQVQHTYFTGSEVENVSVNVHNPTGRSICPSQEPMSIYVYALPLKM   120
Towne       YTPDSTPCHRGDNQLQVQHTYFTGSEVENVSVNVHNPTGRSICPSQEPMSIYVYALPLKM   120
TR          YTPDSTPCHRGDNQLQVQHTYFTGSEVENVSVNVHNPTGRSICPSQEPMSIYVYALPLKM   120
            ************************************************************

Merlin      LNIPSINVHHYPSAAERKHRHLPVADAVIHASGKQMWQARLTVSGLAWTRQQNQWKEPDV   180
TB40/E      LNIPSINVHHYPSAAERKHRHLPVADAVIHASGKQMWQARLTVSGLAWTRQQNQWKEPDV   180
JP          LNIPSINVHHYPSAAERKHRHLPVADAVIHASGKQMWQARLTVSGLAWTRQQNQWKEPDV   180
JHC         LNIPSINVHHYPSAAERKHRHLPVADAVIHASGKQMWQARLTVSGLAWTRQQNQWKEPDV   180
HAN1        LNIPSINVHHYPSAAERKHRHLPVADAVIHASGKQMWQARLTVSGLAWTRQQNQWKEPDV   180
AD169       LNIPSINVHHYPSAAERKHRHLPVADAVIHASGKQMWQARLTVSGLAWTRQQNQWKEPDV   180
Toledo      LNIPSINVHHYPSAAERKHRHLPVADAVIHASGKQMWQARLTVSGLAWTRQQNQWKEPDV   180
Davis       LNIPSINVHHYPSAAERKHRHLPVADAVIHASGKQMWQARLTVSGLAWTRQQNQWKEPDV   180
Towne       LNIPSINVHHYPSAAERKHRHLPVADAVIHASGKQMWQARLTVSGLAWTRQQNQWKEPDV   180
TR          LNIPSINVHHYPSAAERKHRHLPVADAVIHASGKQMWQARLTVSGLAWTRQQNQWKEPDV   180
            ************************************************************

Merlin      YYTSAFVFPTKDVALRHVVCAHELVCSMENTRATKMQVIGDQYVKVYLESFCEDVPSGKL   240
TB40/E      YYTSAFVFPTKDVALRHVVCAHELVCSMENTRATKMQVIGDQYVKVYLESFCEDVPSGKL   240
JP          YYTSAFVFPTKDVALRHVVCAHELVCSMENTRATKMQVIGDQYVKVYLESFCEDVPSGKL   240
JHC         YYTSAFVFPTKDVALRHVVCAHELVCSMENTRATKMQVIGDQYIKVYLESFCEDVPSGKL   240
HAN1        YYTSAFVFPTKDVALRHVVCAHELVCSMENTRATKMQVIGDQYVKVYLESFCEDVPSGKL   240
AD169       YYTSAFVFPTKDVALRHVVCAHELVCSMENTRATKMQVIGDQYVKVYLESFCEDVPSGKL   240
Toledo      YYTSAFVFPTKDVALRHVVCAHELVCSMENTRATKMQVIGDQYVKVYLESFCEDVPSGKL   240
Davis       YYTSAFVFPTKDVALRHVVCAHELVCSMENTRATKMQVIGDQYVKVYLESFCEDVPSGKL   240
Towne       YYTSAFVFPTKDVALRHVVCAHELVCSMENTRATKMQVIGDQYVKVYLESFCEDVPSGKL   240
TR          YYTSAFVFPTKDVALRHVVCAHELVCSMENTRATKMQVIGDQYVKVYLESFCEDVPSGKL   240
            *******************************************:****************

Merlin      FMHVTLGSDVEEDLTMTRNPQPFMRPHERNGFTVLCPKNMIIKPGKISHIMLDVAFTSHE   300
TB40/E      FMHVTLGSDVEEDLTMTRNPQPFMRPHERNGFTVLCPKNMIIKPGKISHIMLDVAFTSHE   300
JP          FMHVTLGSDVEEDLTMTRNPQPFMRPHERNGFTVLCPKNMIIKPGKISHIMLDVAFTSHE   300
JHC         FMHVTLGSDVEEDLTMTRNPQPFMRPHERNGFTVLCPKNMIIKPGKISHIMLDVAFTSHE   300
HAN1        FMHVTLGSDVEEDLTMTRNPQPFMRPHERNGFTVLCPKNMIIKPGKISHIMLDVAFTSHE   300
AD169       FMHVTLGSDVEEDLTMTRNPQPFMRPHERNGFTVLCPKNMIIKPGKISHIMLDVAFTSHE   300
Toledo      FMHVTLGSDVEEDLTMTRNPQPFMRPHERNGFTVLCPKNMIIKPGKISHIMLDVAFTSHE   300
Davis       FMHVTLGSDVEEDLTMTRNPQPFMRPHERNGFTVLCPKNMIIKPGKISHIMLDVAFTSHE   300
Towne       FMHVTLGSDVEEDLTMTRNPQPFMRPHERNGFTVLCPKNMIIKPGKISHIMLDVAFTSHE   300
TR          FMHVTLGSDVEEDLTMTRNPQPFMRPHERNGFTVLCPKNMIIKPGKISHIMLDVAFTSHE   300
            ************************************************************
```

```
Merlin      HFGLLCPKSIPGLSISGNLLMNGQQIFLEVQAIRETVELRQYDPVAALFFFDIDLLLQRG    360
TB40/E      HFGLLCPKSIPGLSISGNLLMNGQQIFLEVQAIRETVELRQYDPVAALFFFDIDLLLQRG    360
JP          HFGLLCPKSIPGLSISGNLLMNGQQIFLEVQAIRETVELRQYDPVAALFFFDIDLLLQRG    360
JHC         HFGLLCPKSIPGLSISGNLLMNGQQIFLEVQAIRETVELRQYDPVAALFFFDIDLLLQRG    360
HAN1        HFGLLCPKSIPGLSISGNLLMNGQQIFLEVQAIRETVELRQYDPVAALFFFDIDLLLQRG    360
AD169       HFGLLCPKSIPGLSISGNLLMNGQQIFLEVQAIRETVELRQYDPVAALFFFDIDLLLQRG    360
Toledo      HFGLLCPKSIPGLSISGNLLMNGQQIFLEVQAIRETVELRQYDPVAALFFFDIDLLLQRG    360
Davis       HFGLLCPKSIPGLSISGNLLMNGQQIFLEVQAIRETVELRQYDPVAALFFFDIDLLLQRG    360
Towne       HFGLLCPKSIPGLSISGNLLMNGQQIFLEVQAIRETVELRQYDPVAALFFFDIDLLLQRG    360
TR          HFGLLCPKSIPGLSISGNLLMNGQQIFLEVQAIRETVELRQYDPVAALFFFDIDLLLQRG    360
            ************************************************************

Merlin      PQYSEHPTFTSQYRIQGKLEYRHTWDRHDEGAAQGDDDVWTSGSDSDEELVTTERKTPRV    420
TB40/E      PQYSEHPTFTSQYRIQGKLEYRHTWDRHDEGAAQGDDDVWTSGSDSDEELVTTERKTPRV    420
JP          PQYSEHPTFTSQYRIQGKLEYRHTWDRHDEGAAQGDDDVWTSGSDSDEELVTTERKTPRV    420
JHC         PQYSEHPTFTSQYRIQGKLEYRHTWDRHDEGAAQGDDDVWTSGSDSDEELVTTERKTPRV    420
HAN1        PQYSEHPTFTNQYRIQCKLEYRHTWDRHDEGAAQGDDDVWTSGSDSDEELVTTERKTPRV    420
AD169       PQYSEHPTFTSQYRIQGKLEYRHTWDRHDEGAAQGDDDVWTSGSDSDEELVTTERKTPRV    420
Toledo      PQYSEHPTFTNQYRIQGKLEYRHTWDRHDEGAAQGDDDVWTSGSDSDEELVTTERKTPRV    420
Davis       PQYSEHPTFTSQYRIQGKLEYRHTWDRHDEGAAQGDDDVWTSGSDSDEELVTTERKTPRV    420
Towne       PQYSEHPTFTSQYRIQGKLEYRHTWDRHDEGAAQGDDDVWTSGSDSDEELVTTERKTPRV    420
TR          PQYSEHPTFTSQYRIQGKLEYRHTWDRHDEGAAQGDDDVWTSGSDSDEELVTTERKTPRV    420
            **********.***** *******************************************

Merlin      TGGGAMASASTSAGRKRKSASSATACTAGVMTRGRLKAESTVAPEEDTDEDSDNEIHNPA    480
TB40/E      TGGGAMAGASTSAGRKRKSASSATACTSGVMTRGRLKAESTVAPEEDTDEDSDNEIHNPA    480
JP          TGGGAMAGASTSAGRKRKSASSATACTAGVMTRGRLKAESTVAPEEDTDEDSDNEIHNPA    480
JHC         TGGGAMAGASTSAGRKRKSASSATACTSGVMTRGRLKAESTVAPEEDTDEDSDNEIHNPA    480
HAN1        TGGGAMAGASTSAGRKRKSASSATACTAGVMTRGRLKAESTVAPEEDTDEDSDNEIHNPA    480
AD169       TGGGAMAGASTSAGRKRKSASSATACTSGVMTRGRLKAESTVAPEEDTDEDSDNEIHNPA    480
Toledo      TGGGAMAGASTSAGRKRKSASSATACTAGVMTRGRLKAESTVAPEEDTDEDSDNEIHNPA    480
Davis       TGGGAMAGASTSAGRKRKSASSATACTSGVMTRGRLKAESTVAPEEDTDEDSDNEIHNPA    480
Towne       TGGGAMAGASTSAGRKRKSASSATACTAGVMTRGRLKAESTVAPEEDTDEDSDNEIHNPA    480
TR          TGGGAMAGASTSAGRKRKSASSATACTAGVMTRGRLKAESTVAPEEDTDEDSDNEIHNPA    480
            *******.*******************:********************************

Merlin      VFTWPPWQAGILARNLVPMVATVQGQNLKYQEFFWDANDIYRIFAELEGVWQPAAQPKRR    540
TB40/E      VFTWPPWQAGILARNLVPMVATVQGQNLKYQEFFWDANDIYRIFAELEGVWQPAAQPKRR    540
JP          VFTWPPWQAGILARNLVPMVATVQGQNLKYQEFFWDANDIYRIFAELEGVWQPAAQPKRR    540
JHC         VFTWPPWQAGILARNLVPMVATVQGQNLKYQEFFWDANDIYRIFAELEGVWQPAAQPKRR    540
HAN1        VFTWPPWQAGILARNLVPMVATVQGQNLKYQEFFWDANDIYRIFAELEGVWQPAAQPKRR    540
AD169       VFTWPPWQAGILARNLVPMVATVQGQNLKYQEFFWDANDIYRIFAELEGVWQPAAQPKRR    540
Toledo      VFTWPPWQAGILARNLVPMVATVQGQNLKYQEFFWDANDIYRIFAELEGVWQPAAQPKRR    540
Davis       VFTWPPWQAGILARNLVPMVATVQGQNLKYQEFFWDANDIYRIFAELEGVWQPAAQPKRR    540
Towne       VFTWPPWQAGILARNLVPMVATVQGQNLKYQEFFWDANDIYRIFAELEGVWQPAAQPKRR    540
TR          VFTWPPWQAGILARNLVPMVATVQGQNLKYQEFFWDANDIYRIFAELEGVWQPAAQPKRR    540
            ************************************************************

Merlin      RHRQDALPGPCIASTPKKHRG                                           561
TB40/E      RHRQEALPGPCIASTPKKHRG                                           561
JP          RHRQEALPGPCIASTPKKHRG                                           561
JHC         RHRQDALPGPCIASTPKKHRG                                           561
HAN1        RHRQDALPGPCIASTPKKHRG                                           561
AD169       RHRQDALPGPCIASTPKKHRG                                           561
Toledo      RHRQEALPGPCIASTPKKHRG                                           561
Davis       RHRQEALPGPCIASTPKKHRG                                           561
Towne       RHRQDALPGPCIASTPKKHRG                                           561
TR          RHRQEALPGPCIASTPKKHRG                                           561
            ****:****************
```

iii)

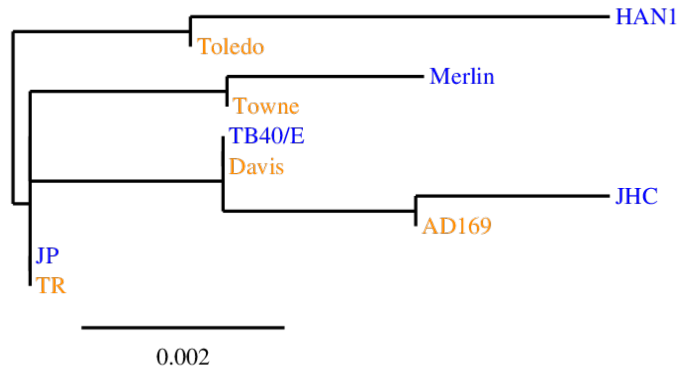

HAN1
Toledo
Merlin
Towne
TB40/E
Davis
JHC
AD169
JP
TR
0.002

iv)

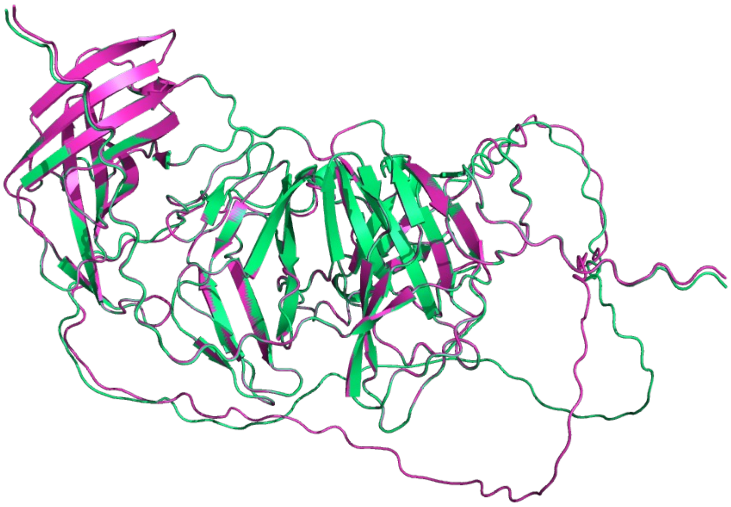

v)

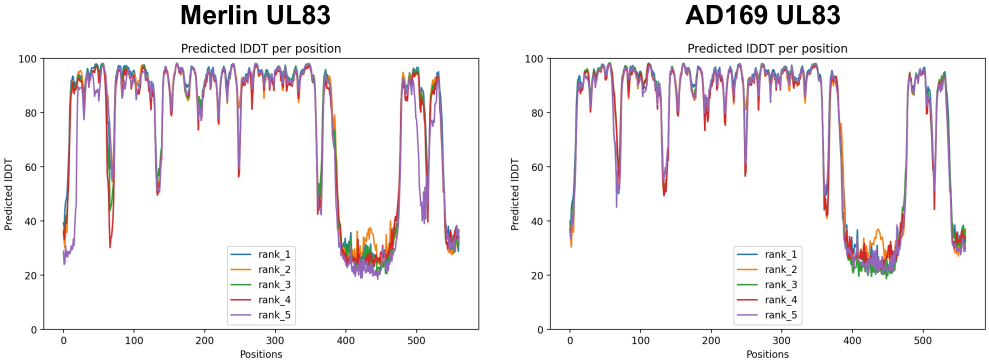

Merlin UL83
Predicted lDDT per position
Predicted lDDT
Positions
rank_1
rank_2
rank_3
rank_4
rank_5
AD169 UL83
Predicted lDDT per position
Predicted lDDT
Positions
rank_1
rank_2
rank_3
rank_4
rank_5

F- UL122

i)

```
Merlin    MESSAKRKMDPDNPDEGPSSKVPRPETPVTKATTFLQTMLRKEVNSQLSLGDPLFPELAE   60
AD169     MESSAKRKMDPDNPDEGPSSKVPRPETPVTKATTFLQTMLRKEVNSQLSLGDPLFPELAE   60
          ************************************************************

Merlin    ESLKTFEQVTEDCNENPEKDVLTELGDILAQAVNHAGIDSSSTGPTLTTHSCSVSSAPLN   120
AD169     ESLKTFEQVTEDCNENPEKDVLAELGDILAQAVNHAGIDSSSTGPTLTTHSCSVSSAPLN   120
          **********************:*************************************

Merlin    KPTPTSVAVTNTPLPGASATPELSPRKKPRKTTRPFKVIIKPPVPPAPIMLPLIKQEDIK   180
AD169     KPTPTSVAVTNTPLPGASATPELSPRKKPRKTTRPFKVIIKPPVPPAPIMLPLIKQEDIK   180
          ************************************************************

Merlin    PEPDFTIQYRNKIIDTAGCIVISDSEEEQGEEVETRGATASSPSTGSGTPRVTSPTHPLS   240
AD169     PEPDFTIQYRNKIIDTAGCIVISDSEEEQGEEVETRGATASSPSTGSGTPRVTSPTHPLS   240
          ************************************************************

Merlin    QMNHPPLPDPLGRPDEDSSSSSSSSCSSASDSESESEEMKCSSGGGASVTSSHHGRGGFG   300
AD169     QMNHPPLPDPLGRPDEDSSSSSSSSCSSASDSESESEEMKCSSGGGASVTSSHHGRGGFG   300
          ************************************************************

Merlin    GAASSSLLSCGHQSSGGASTGPRKKKSKRISELDNEKVRNIMKDKNTPFCTPNVQTRRGR   360
AD169     GAASSSLLSCGHQSSGGASTGPRKKKSKRISELDNEKVRNIMKDKNTPFCTPNVQTRRGR   360
          ************************************************************

Merlin    VKIDEVSRMFRHTNRSLEYKNLPFMIPSMHQVLEEAIKVCKTMQVNNKGIQIIYTRNHEV   420
AD169     VKIDEVSRMFRNTNRSLEYKNLPFTIPSMHQVLDEAIKACKTMQVNNKGIQIIYTRNHEV   420
          ***********:************ ********:****.*********************

Merlin    KNEVDQVRCRLGSMCNLALSTPFLMEHTMPVTHPPDVAQRTADACNDGVKAVWNLKELHT   480
AD169     KSEVDAVRCRLGTMCNLALSTPFLMEHTMPVTHPPEVAQRTADACNEGVKAAWSLKELHT   480
          *.*** ******:**********************:**********:****.*.******

Merlin    HQLCPRSSDYRNMIIHAATPVDLLGALNLCLPLMQKFPKQVMVRIFSTNQGGFMLPIYET   540
AD169     HQLCPRSSDYRNMIIHAATPVDLLGALNLCLPLMQKFPKQVMVRIFSTNQGGFMLPIYET   540
          ************************************************************

Merlin    AAKAYAVGQFEKPTETPPEDLDTLSLAIEAAIQDLRNKSQ                     580
AD169     AAKAYAVGQFEQPTETPPEDLDTLSLAIEAAIQDLRNKSQ                     580
          ***********:****************************
```

ii)

```
Merlin      MESSAKRKMDPDNPDEGPSSKVPRPETPVTKATTFLQTMLRKEVNSQLSLGDPLFPELAE   60
TB40/E      MESSAKRKMDPDNPDEGPSSKVPRPETPVTKATTFLQTMLRKEVNSQLSLGDPLFPELAE   60
JP          MESSAKRKMDPDNPDEGPSSKVPRPETPVTKATTFLQTMLRKEVNSQLSLGDPLFPELAE   60
JHC         MESSAKRKMDPDNPDEGPSSKVPRPETPVSKATTFLQTMLRKEVNSQLSLGDPLFPELAE   60
HAN1        MESSAKRKMDPDNPDEGPSSKVPRPETPVTKATTFLQTMLRKEVNSQLSLGDPLFPELAE   60
AD169       MESSAKRKMDPDNPDEGPSSKVPRPETPVTKATTFLQTMLRKEVNSQLSLGDPLFPELAE   60
Toledo      MESSAKRKMDPDNPDEGPSSKVPRPETPVTKATTFLQTMLRKEVNSQLSLGDPLFPELAE   60
Davis       MESSAKRKMDPDNPDEGPSSKVPRPETPVTKATTFLQTMLRKEVNSQLSLGDPLFPELAE   60
Towne       MESSAKRKMDPDNPDEGPSSKVPRPETPVTKATTFLQTMLRKEVNSQLSLGDPLFPELAE   60
TR          MESSAKRKMDPDNPDEGPSSKVPRPETPVTKATTFLQTMLRKEVNSQLSLGDPLFPELAE   60
            *****************************:******************************

Merlin      ESLKTFEQVTEDCNENPEKDVLTELGDILAQAVNHAGIDSSSTGPTLTTHSCSVSSAPLN   120
TB40/E      ESLKTFEQVTEDCNENPEKDVLAELGDILAQAVNHAGIDSSSTGPTLTTHSCSVSSAPLN   120
JP          ESLKTFEQVTEDCNENPEKDVLAELGDILAQAVNHAGIDSSSTGPTLTTHSCSVSSAPLN   120
JHC         ESLKTFEQVTEDCNENPEKDVLAELGDILAQAVNHAGIDSSSTGPTLTTHSCSVSSAPLN   120
HAN1        ESLKTFEQVTEDCNENPEKDVLAELGDILAQAVNHAGIDSSSTGPTLTTHSCSVSSAPLN   120
AD169       ESLKTFEQVTEDCNENPEKDVLAELGDILAQAVNHAGIDSSSTGPTLTTHSCSVSSAPLN   120
Toledo      ESLKTFEQVTEDCNENPEKDVLAELGDILAQAVNHAGIDSSSTGPTLTTHSCSVSSAPLN   120
Davis       ESLKTFEQVTEDCNENPEKDVLAELGDILAQAVNHAGIDSSSTGPTLTTHSCSVSSAPLN   120
Towne       ESLKTFERVTEDCNENPEKDVLAELGDILAQAVNHAGIDSSSTGPTLTTHSCSVSSAPLN   120
TR          ESLKTFEQVTEDCNENPEKDVLAELGDILAQAVNHAGIDSSSTGHTLTTHSCSVSSAPLN   120
            *******:**************:********************* ***************

Merlin      KPTPTSVAVTNTPLPGASATPELSPRKKPRKTTRPFKVIIKPPVPPAPIMLPL-IKQEDI   179
TB40/E      KPTPTSVAVTNTPLPGASATPELSPRKKPRKTTRPFKVIIKPPVPPAPIMLPL-IKQEDI   179
JP          KPTPTSVAVTNTPLPGASATPELSPRKKPRKTTRPFKVIIKPPVPPAPIMLPL-IKQEDI   179
JHC         KPTPTSVAVTNTPIPGASATPELSPRKKPRKTTRPFKVIIKPPVPPAPIMLPL-IKQEDI   179
HAN1        KPTPTSVAVTNTPLPGASATPELSPRKKPRKTTRPFKVIIKPPVPPAPIMLPL-IKQEDI   179
AD169       KPTPTSVAVTNTPLPGASATPELSPRKKPRKTTRPFKVIIKPPVPPAPIMLPL-IKQEDI   179
Toledo      KPTPTSVAVTNTPLPGASATPELSPRKKPRKTTRPFKVIIKPPVPPAPIMLPL-IKQEDI   179
Davis       KPTPTSVAVTNTPLPGASATPELSPRKKPRKTTRPFKVIIKPPVPPAPIMLPL-IKQEDI   179
Towne       KPTPTSVAVTNTPLPGASATPELSPRKKPRKTTRPFKVIIKPPVPPAPIMLPL-IKQEDI   179
TR          KPTPTSVAVTNTPLPGASATPELSPRKKPRKTTRPFKVIIKPPVPPAPIMLPLLIKQEDI   180
            *************:*************************************** ******

Merlin      KPEPDFTIQYRNKIIDTAGCIVISDSEEEQGEEVETRGATASSPSTGSGTPRVTSPTHPL   239
TB40/E      KPEPDFTIQYRNKIIDTAGCIVISDSEEEQGEEVETRGATASSPSTGSGTPRVTSPTHPL   239
JP          KPEPDFTIQYRNKIIDTAGCIVISDSEEEQGEEVETRGATASSPSTGSGTPRVTSPTHPL   239
JHC         KPEPDFTIQYRNKIIDTAGCIVISDSEEEQGEEVETRGATASSPSTGSGTPRVTSPTHPL   239
HAN1        KPEPDFTIQYRNKIIDTAGCIVISDSEEEQGEEVETRGATASSPSTGSGTPRVTSPTHPL   239
AD169       KPEPDFTIQYRNKIIDTAGCIVISDSEEEQGEEVETRGATASSPSTGSGTPRVTSPTHPL   239
Toledo      KPEPDFTIQYRNKIIDTAGCIVISDSEEEQGEEVETRGATASSPSTGSGTPRVTSPTHPL   239
Davis       KPEPDFTIQYRNKIIDTAGCIVISDSEEEQGEEVETRGATASSPSTGSGTPRVTSPTHPL   239
Towne       KPEPDFTIQYRNKIIDTAGCIVISDSEEEQGEEVETRGATASSPSTGSGTPRVTSPTHPL   239
TR          KPEPDFTIQYRNKIIDTAGCIVISDSEEEQGEEVETRGATASSPSTGSGTPRVTSPTHPL   240
            ************************************************************

Merlin      SQMNHPPLPDPLGRPDEDSSSSSSSSSCSSASDSESESEEMKCSSGGGASVTSSHHGRGGF   299
TB40/E      SQMNHPPLPDPLGRPDEDSSSSSSSSSCSSASDSESESEEMKCSSGGGASVTSSHHGRGGF   299
JP          SQMNHPPLPDPLGRPDEDSSSSSSSSSCSSASDSESESEEMKCSSGGGASVTSSHHGRGGF   299
JHC         SQMNHPPLPDPLARPDEDSSSSSSSSSCSSASDSESESEEMKCSSGGGASVTSSHHGRGGF   299
HAN1        SQMNHPPLPDPLGRPDEDSSSSSSSSSCSSASDSESESEEMKCSSGGGASVTSSHHGRGGF   299
AD169       SQMNHPPLPDPLGRPDEDSSSSSSSSSCSSASDSESESEEMKCSSGGGASVTSSHHGRGGF   299
Toledo      SQMNHPPLPDPLARPDEDSSSSSSSSSCSSASDSESESEEMKCSSGGGASVTSSHHGRGGF   299
Davis       SQMNHPPLPDPLGRPDEDSSSSSSSSSCSSASDSESESEEMKCSSGGGASVTSSHHGRGGF   299
Towne       SQMNHPPLPDPLGRPDEDSSS-SSSSCSSASDSESESEEMKCSSGGGASVTSSHHGRGGF   298
TR          SQMNHPPLPDPLGRPDEDSSSSSSSSSCSSASDSESESEEMKCSSGGGASVTSSHHGRGGF   300
            ************.******** ***************************************
```

```
Merlin      GGAASSSLLSCGHQSSGGASTGPRKKKSKRISELDNEKVRNIMKDKNTPFCTPNVQTRRG   359
TB40/E      GGAASSSLLSCGHQSSGGASTGPRKKKSKRISELDNEKVRNIMKDKNTPFCTPNVQTRRG   359
JP          GGAASSSLLSCGHQSSGGASTGPRKKKSKRISELDNEKVRNIMKDKNTPFCTPNVQTRRG   359
JHC         GGAASSSLLSCGHQSSGGASTGPRKKKSKRISELDNEKVRNIMKDKNTPFCTPNVQTRRG   359
HAN1        GGAASSSLLSCGHQSSGGASTGPRKKKSKRISELDNEKVRNIMKDKNTPFCTPNVQTRRG   359
AD169       GGAASSSLLSCGHQSSGGASTGPRKKKSKRISELDNEKVRNIMKDKNTPFCTPNVQTRRG   359
Toledo      GGAASSSLLSCGHQSSGGASTGPRKKKSKRISELDNEKVRNIMKDKNTPFCTPNVQTRRG   359
Davis       GGAASSSLLSCGHQSSGGASTGPRKKKSKRISELDNEKVRNIMKDKNTPFCTPNVQTRRG   359
Towne       GGAASSSLLSCGHQSSGGASTGPRKKKSKRISELDNEKVRNIMKDKNTPFCTPNVQTRRG   358
TR          GGAASSSLLSCGHQSSGGASTGPRKKKSKRISELDNEKVRNIMKDKNTPFCTPNVQTRRG   360
            ************************************************************

Merlin      RVKIDEVSRMFRHTNRSLEYKNLPFMIPSMHQVLEEAIKVCKTMQVNNKGIQIIYTRNHE   419
TB40/E      RVKIDEVSRMFRNTNRSLEYKNLPFTIPSMDQVLDEAIKACKTMQVNNKGIQIIYTRNHE   419
JP          RVKIDEVSRMFRNTNRSLEYKNLPFMIPSMHQVLEEAIKACKTMQVNNKGIQIIYTRNHE   419
JHC         RVKIDEVSRMFRNTNRSLEYKNLPFTIPSMHQVLDEAIKACKTMQVNNKGIQIIYTRNHE   419
HAN1        RVKIDEVSRMFRNTNRSLEYKNLPFTIPSMHQVLDEAIKACKTMQVNNKGIQIIYTRNHE   419
AD169       RVKIDEVSRMFRNTNRSLEYKNLPFTIPSMHQVLDEAIKACKTMQVNNKGIQIIYTRNHE   419
Toledo      RVKIDEVSRMFRNTNRSLEYKNLPFTIPSMHQVLDEAIKACKTMQVNNKGIQIIYTRNHE   419
Davis       RVKIDEVSRMFRNTNRSLEYKNLPFTIPSMHQVLDEAIKACKTMQVNNKGIQIIYTRNHE   419
Towne       RVKIDEVSRMFRNTNRSLEYKNLPFTIPSMHQVLDEAIKACKTMQVNNKGIQIIYTRNHE   418
TR          RVKIDEVSRMFRNTNRSLEYKNLPFMIPSMHQVLDEAIKACKTMQVNNKGIQIIYTRNHE   420
            ************:************ ****.***:****.********************

Merlin      VKNEVDQVRCRLGSMCNLALSTPFLMEHTMPVTHPPDVAQRTADACNDGVKAVWNLKELH   479
TB40/E      VKSEVDAVRCRLGTMCNLALSTPFLMEHTMPVTHPPEVAQRTADACNEGVKAAWSLKELH   479
JP          VKNEVDQVRCRLGTMCNLALSTPFLMEHTMPVTHPPEVAQRTADACNEGVKAAWSLKELH   479
JHC         VKSEVDAVRCRLGTMCNLALSTPFLMEHTMPVTHPPEVAQRTADACNEGVKAAWSLKELH   479
HAN1        VKSEVDAVRCRLGTMCNLALSTPFLMEHTMPVTHPPEVAQRTADACNEGVKAAWSLKELH   479
AD169       VKSEVDAVRCRLGTMCNLALSTPFLMEHTMPVTHPPEVAQRTADACNEGVKAAWSLKELH   479
Toledo      VKSEVDAVRCRLGTMCNLALSTPFLMEHTMPVTHPPEVAQRTADACNEGVKAAWSLKELH   479
Davis       VKSEVDAVRCRLGTMCNLALSTPFLMEHTMPVTHPPEVAQRTADACNEGVKAAWSLKELH   479
Towne       VKSEVDAVRCRLGTMCNLALSTPFLMEHTMPVTHPPEVAQRTADACNEGVKAAWSLKELH   478
TR          VKSEVDAVRCRLGTMCNLALSTPFLMEHTMPVTHPPEVAQRTADACNEGVKAAWSLKELH   480
            **.*** ******:**********************:**********:****.*.*****

Merlin      THQLCPRSSDYRNMIIHAATPVDLLGALNLCLPLMQKFPKQVMVRIFSTNQGGFMLPIYE   539
TB40/E      THQLCPRSSDYRNMIIHAATPVDLLGALNLCLPLMQKFPKQVMVRIFSTNQGGFMLPIYE   539
JP          THQLCPRSSDYRNMIIHAATPVDLLGALNLCLPLMQKFPKQVMVRIFSTNQGGFMLPIYE   539
JHC         THQLCPRSSDYRNMIIHAATPVDLLGALNLCLPLMQKFPKQVMVRIFSTNQGGFMLPIYE   539
HAN1        THQLCPRSSDYRNMIIHAATPVDLLGALNLCLPLMQKFPKQVMVRIFSTNQGGFMLPIYE   539
AD169       THQLCPRSSDYRNMIIHAATPVDLLGALNLCLPLMQKFPKQVMVRIFSTNQGGFMLPIYE   539
Toledo      THQLCPRSSDYRNMIIHAATPVDLLGALNLCLPLMQKFPKQVMVRIFSTNQGGFMLPIYE   539
Davis       THQLCPRSSDYRNMIIHAATPVDLLGALNLCLPLMQKFPKQVMVRIFSTNQGGFMLPIYE   539
Towne       THQLCPRSSDYRNMIIHAATPVDLLGALNLCLPLMQKFPKQVMVRIFSTNQGGFMLPIYE   538
TR          THQLCPRSSDYRNMIIHAATPVDLLGALNLCLPLMQKFPKQVMVRIFSTNQGGFMLPIYE   540
            ************************************************************

Merlin      TAAKAYAVGQFEKPTETPPEDLDTLSLAIEAAIQDLRNKSQ                      580
TB40/E      TAAKAYAVGQFEQPTETPPEDLDTLSLAIEAAIQDLRNKSQ                      580
JP          TAAKAYAVGQFEQPTETPPEDLDTLSLAIEAAIQDLRNKSQ                      580
JHC         TAAKAYAVGQFEQPTETPPEDLDTLSLAIEAAIQDLRNKSQ                      580
HAN1        TAAKAYAVGQFEQPTETPPEDLDTLSLAIEAAIQDLRNKSQ                      580
AD169       TAAKAYAVGQFEQPTETPPEDLDTLSLAIEAAIQDLRNKSQ                      580
Toledo      TAAKAYAVGQFEQPTETPPEDLDTLSLAIEAAIQDLRNKSQ                      580
Davis       TAAKAYAVEQFEQPTETPPEDLDTLSLAIEAAIQDLRNKSQ                      580
Towne       TAAKAYAVGQFEQPTETPPEDLDTLSLAIEAAIQDLRNKSQ                      579
TR          TAAKAYDVGQFEQPTETPPEDLDTLSLAIEAAIQDLRNKSQ                      581
            ****** * ***:****************************
```

iii)

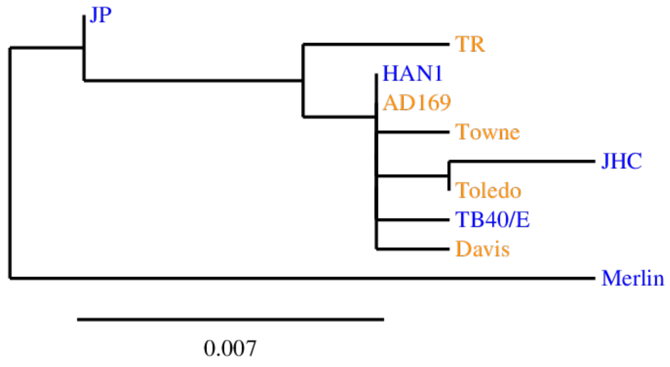
JP
TR
HAN1
AD169
Towne
JHC
Toledo
TB40/E
Davis
Merlin
0.007

iv)

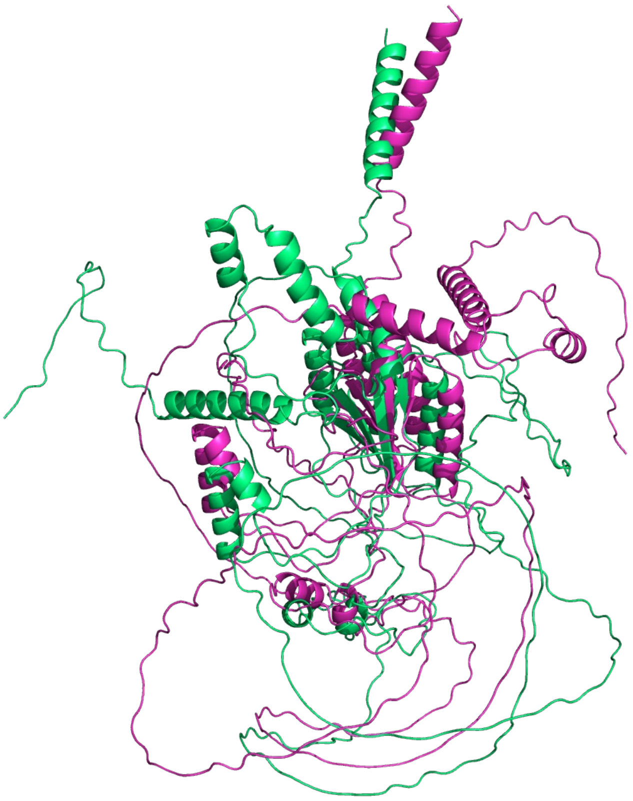

v)

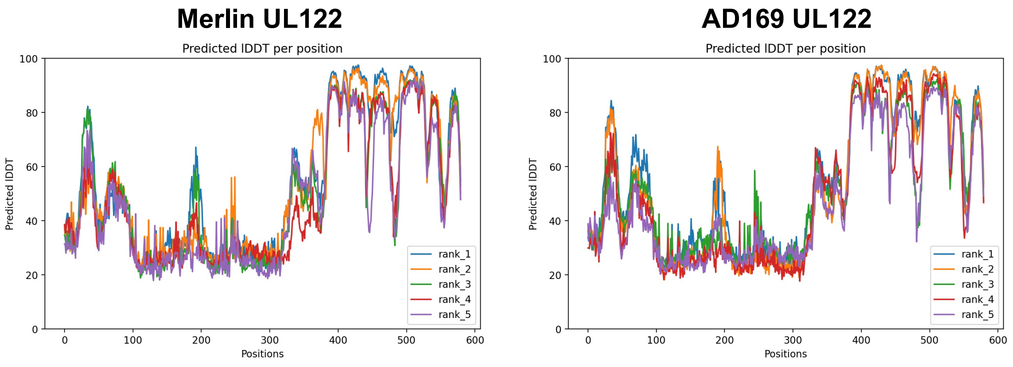
Merlin UL122
Predicted lDDT per position
Predicted lDDT
Positions
rank_1
rank_2
rank_3
rank_4
rank_5
AD169 UL122
Predicted lDDT per position
Predicted lDDT
Positions
rank_1
rank_2
rank_3
rank_4
rank_5

G- UL123

ii)

```
Merlin      MESSAKRKMDPDNPDEGPSSKVPRPETPVTKATTFLQTMLRKEVNSQLSLGDPLFPELAE    60
TB40/E      MESSAKRKMDPDNPDEGPSSKVPRPETPVTKATTFLQTMLRKEVNSQLSLGDPLFPELAE    60
JP          MESSAKRKMDPDNPDEGPSSKVPRPETPVTKATTFLQTMLRKEVNSQLSLGDPLFPELAE    60
JHC         MESSAKRKMDPDNPDEGPSSKVPRPETPVSKATTFLQTMLRKEVNSQLSLGDPLFPELAE    60
HAN1        MESSAKRKMDPDNPDEGPSSKVPRPETPVTKATTFLQTMLRKEVNSQLSLGDPLFPELAE    60
AD169       MESSAKRKMDPDNPDEGPSSKVPRPETPVTKATTFLQTMLRKEVNSQLSLGDPLFPELAE    60
Toledo      MESSAKRKMDPDNPDEGPSSKVPRPETPVTKATTFLQTMLRKEVNSQLSLGDPLFPELAE    60
Davis       MESSAKRKMDPDNPDEGPSSKVPRPETPVTKATTFLQTMLRKEVNSQLSLGDPLFPELAE    60
Towne       MESSAKRKMDPDNPDEGPSSKVPRPETPVTKATTFLQTMLRKEVNSQLSLGDPLFPELAE    60
TR          MESSAKRKMDPDNPDEGPSSKVPRPETPVTKATTFLQTMLRKEVNSQLSLGDPLFPELAE    60
            *****************************:******************************

Merlin      ESLKTFEQVTEDCNENPEKDVLTELVKQIKVRVDMVRHRIKEHMLKKYTQTEEKFTGAFN    120
TB40/E      ESLKTFEQVTEDCNENPEKDVLAELVKQIKVRVDMVRHRIKEHMLKKYTQTEEKFTGAFN    120
JP          ESLKTFEQVTEDCNENPEKDVLAELVKQIKVRVDMVRHRIKEHMLKKYTQTEEKFTGAFN    120
JHC         ESLKTFEQVTEDCNENPEKDVLAELVKQIKVRVDMVRHRIKEHMLKKYTQTEEKFTGAFN    120
HAN1        ESLKTFEQVTEDCNENPEKDVLAELVKQIKVRVDMVRHRIKEHMLKKYTQTEEKFTGAFN    120
AD169       ESLKTFEQVTEDCNENPEKDVLAELVKQIKVRVDMVRHRIKEHMLKKYTQTEEKFTGAFN    120
Toledo      ESLKTFEQVTEDCNENPEKDVLAELVKQIKVRVDMVRHRIKEHMLKKYTQTEEKFTGAFN    120
Davis       ESLKTFEQVTEDCNENPEKDVLAELVKQIKVRVDMVRHRIKEHMLKKYTQTEEKFTGAFN    120
Towne       ESLKTFERVTEDCNENPEKDVLAELVKQIKVRVDMVRHRIKEHMLKKYTQTEEKFTGAFN    120
TR          ESLKTFEQVTEDCNENPEKDVLAELVKQIKVRVDMVRHRIKEHMLKKYTQTEEKFTGAFN    120
            *******:**************:*************************************

Merlin      MMGGCLQNALDILDKVHEPFEDMKCIGLTMQSMYENYIVPEDKREMWMACIKELHDVSKG    180
TB40/E      MMGGCLQNALDILDKVHEPFEEMKCIGLTMQSMYENYIVPEDKREMWMACIKELHDVTKG    180
JP          MMGGCLQNALDILDKVHEPFEEMKCIGLTMQSMYENYIVPEDKREMWMACIKELHDVSKG    180
JHC         MMGGCLQNALDILDKVHEPFEEMKCIGLTMQSMYENYIVPEDKREMWMACIKELHDVTKG    180
HAN1        MMGGCLQNALDILDKVHEPFEEMKCIGLTMQSMYENYIVPEDKREMWMACIKELHDVTKG    180
AD169       MMGGCLQNALDILDKVHEPFEEMKCIGLTMQSMYENYIVPEDKREMWMACIKELHDVSKG    180
Toledo      MMGGCLQNALDILDKVHEPFEEMKCIGLTMQNMYENYIVPEDKREMWMACIKELYNVTKG    180
Davis       MMGGCLQNALDILDKVHEPFEEMKCIGLTMQSMYENYIVPEDKREMWMACIKELHDVTKG    180
Towne       MMGGCLQNALDILDKVHEPFEEMKCIGLTMQSMYENYIVPEDKREMWMACIKELHDVSKG    180
TR          MMGGCLQNALDILDKVHEPFEEMKCIGLTMQSMYENYIVPEDKREMWMACIKELHDVTKG    180
            *********************:*********.**********************::*:**

Merlin      AANKLGGALQAKARAKKDELRRKMMYMCYRNIEFFTKNSAFPKTTNGCSQAMAALQNLPQ    240
TB40/E      AANKLGGALKAKARAKKDELKRKMIYMCYRNIEFFTKNSAFPKTTNGCSQAMAALQNLPQ    240
JP          AANKLGGALQAKARAKKDELRRKMMYMCYRNIEFFTKNSAFPKTTNGCSQAMAALQNLPQ    240
JHC         AANKLGGALKAKARAKKDELKRKMIYMCYRNVEFFTKNSAFPKTTNGCSQAMAALQNLSQ    240
HAN1        AANKLGGALKAKARAKKDELKRKMIYMCYRNVEFFTKNSAFPKTTNGCSQAMAALQNLPQ    240
AD169       AANKLGGALQAKARAKKDELRRKMMYMCYRNIEFFTKNSAFPKTTNGCSQAMAALQNLPQ    240
Toledo      AANKLGGALQAKARAKKDELKRKMMYMCYRNVEFFTKNSAFPKTTNGCSQAMAALQNLPQ    240
Davis       AANKLGGALKAKARAKKDELKRKMIYMCYRNVEFFTKNSAFPKTTNGCSQAMAALQNLPQ    240
Towne       AANKLGGALQAKARAKKDELRRKMMYMCYRNIEFFTKNSAFPKTTNGCSQAMAALQNLPQ    240
TR          AANKLGGALKAKARAKKDELKRKMIYMCYRNIEFFTKNSAFPKTTNGCSQAMAALQNLPQ    240
            *********:**********:***:******:************************** *

Merlin      CSPDEIMSYAQKIFKILDEERDKVLTHIDHIFMDILTTCVETMCNEYKVTSDACMMTMYG    300
TB40/E      CSPDEIMAYAQKIFKILDEERDKVLTHIDHIFMDILTTCVETMCNEYKVTSDACMMTMYG    300
JP          CSPDEIMAYAQKIFKILDEERDKVLTHIDHIFMDILTTCVETMCNEYKVTSDACMMTMYG    300
JHC         CSPDEIMAYAQKIFKILDEERDKVLTHIDHIFMDILTTCVETMCNEYKVTSDACMMTMYG    300
HAN1        CSPDEIMAYAQKIFKILDEERDKVLTHIDHIFMDILTTCVETMCNEYKVTSDACMMTMYG    300
AD169       CSPDEIMAYAQKIFKILDEERDKVLTHIDHIFMDILTTCVETMCNEYKVTSDACMMTMYG    300
Toledo      CSPNEIMTYSQKIFKILDEERDKVLTHIDHIFMDILTTCVETMCSEYKVTSDACMMGMYG    300
Davis       CSPDEIMAYAQKIFKILDEERDKVLTHIDHIFMDILTTCVETMCNEYKVTSDACMMTMYG    300
Towne       CSPDEIMAYAQKIFKILDEERDKVLTHIDHIFMDILTTCVETMCNEYKVTSDACMMTMYG    300
TR          CSPDEIIAYAQKIFKILDEERDKVLTHIDHIFMDILTTCVETMCNEYKVTSDACMMTMYG    300
            ***:**::*:**********************************.*********** ***
```

```
Merlin      GISLLSEFCRVLCCYVLEETSVMLAKRPLITKPEVISVMKRRIEEICMKVFAQYILGADP    360
TB40/E      SISLLSEFCRVLCCYILEETSVMLAKRPLITKPEVINVMKRRIEEICMKVFAQYILGADP    360
JP          GISLLSEFCRVLCCYVLEETSVMLAKRPLITKPEVISVMKRRIEEICMKVFAQYILGADP    360
JHC         SISLLSEFCRVLCCYILEETSVMMAKRPLITKPEVINIMKRRIEEICMKVFAQYILGADP    360
HAN1        SISLLSEFCRVLCCYILEETSVMLAKRPLITKPEVISVMKRRIEEICMKVFAQYILGADP    360
AD169       GISLLSEFCRVLCCYVLEETSVMLAKRPLITKPEVISVMKRRIEEICMKVFAQYILGADP    360
Toledo      AISLLTEFCRVLCCYILEETSVLLAKRPLITKPEVINIMKRRIEEICMKVFAQYILGADP    360
Davis       SISLLSEFCRVLCCYILEETSVMLAKRPLITKPEVISVMKRRIEEICMKVFAQYILGADP    360
Towne       GISLLSEFCRVLCCYVLEETSVMLAKRPLITKPEVISVMKRRIEEICMKVFAQYILGADP    360
TR          SISLLSEFCRVLCCYILEETSVMLAKRPLITKPEVINVMKRRIEEICMKVFAQYILGADP    360
            .****:*********:******::************.:**********************

Merlin      LRVCSPSVDDLRAIAEESDEEEAIVAYTLATAGASSSDSLVSPPESPVPATIPLSSVIVA    420
TB40/E      LRVCSPSVDDLRAIAEESDEEDAIAAYTLATAGASSSDSLVSPPESPVPATIPLSSVIVA    420
JP          LRVCSPSVDDLRAIAEESDEEEAIVAYTLATAGASSSDSLVSPPESPVPATIPLSSVIVA    420
JHC         LRVCSPSVDDLRAIAEESDEEDAIVAYTLATAGASSSDSLVSPPESPVPATIPLSSVIVA    420
HAN1        LRVCSPSVDDLRAIAEESDEEDAIVAYTLATAGASSSDSLVSPPESPVPATIPLSSVIVA    420
AD169       LRVCSPSVDDLRAIAEESDEEEAIVAYTLATAGVSSSDSLVSPPESPVPATIPLSSVIVA    420
Toledo      LRVCSPSVDDLRAIAEESDEENAIAAYTLATAGASSSDSLVSPPESPVPATIPLSSVIVA    420
Davis       LRVCSPSVDDLRAIAEESDEEDAIAAYTLATAGASSSDSLVSPPESPVPATIPLSSVIVA    420
Towne       LRVCSPSVDDLRAIAEESDEEEAIVAYTLATAGASSSDSLVSPPESPVPATIPLSSVIVA    420
TR          LRVCSPSVDDLRAIAEESDEEDAIAAYTLATAGASSSDSLVSPPESPVPATIPLSSVIVA    420
            *********************:**.********.**************************

Merlin      ENSDQEESEQSDEEQEEGAQEEREDTVSVKSEPVSEIEEVASEEEEDGAEEPTASGGKST    480
TB40/E      ENSDQEESEQSDEEQEEGAQEEQEDTVSVKSEPVSEIEEVASEEEEDGAEEPTTSGGKST    480
JP          ENSDQEESEQSEE-EEEGAQEEREDTVSVKSEPVSEIEEVAPEEEEDGAEEPTASGGKST    479
JHC         ENSDQEESEQSDEEQEEGAQEEREDTVSVKSEPVSEIEEVASEKEEDGAEEPTTSGGKST    480
HAN1        ENSDQEESEQSDEEQEEGAQEEREDTVSVKSEPVSEIEEVASEEEEDGAEEPTTSGGKST    480
AD169       ENSDQEESEQSDEEEEEGAQEEREDTVSVKSEPVSEIEEVAPEEEEDGAEEPTASGGKST    480
Toledo      ENSDQEESEQSDEEREEGAQEEREDTVSVKSEPVSEIEQVASEEEEDGAEEPTTSGGKST    480
Davis       ENSDQEESEQSDEEQEEGAQEEREDTVSVKSEPVSEIEEVASEEEEDGAEEPTTSGGKST    480
Towne       ENSDQEESEQSDEEEEEGAQEEREDTVSVKSEPVSEIEEVAPEEEEDGAEEPTASGGKST    480
TR          ENSDQEESEQSDEEQGEGAQEEREDTVSVKSEPVSEIGEVASEEEEDGAEEPTTSGGKST    480
            ***********:* . ******:************** :** *:*********:******

Merlin      HPMVTRSKADQ                                                     491
TB40/E      HPMVTRSKADQ                                                     491
JP          HPMVTRSKADQ                                                     490
JHC         HPMVTRSKADQ                                                     491
HAN1        HPMVTRSKADQ                                                     491
AD169       HPMVTRSKADQ                                                     491
Toledo      HPMVTRSKADH                                                     491
Davis       HPMVTRSKADQ                                                     491
Towne       HPMVTRSKADQ                                                     491
TR          HPMVTRSKADQ                                                     491
            **********:
```

Supplementary Figure 2

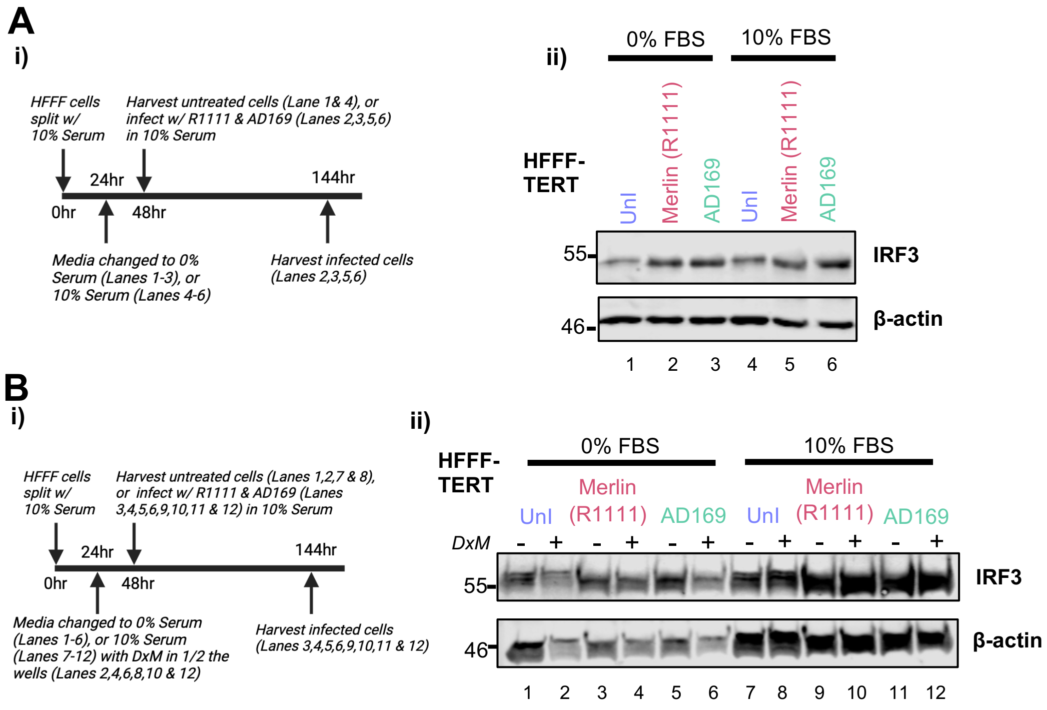

Supplement: Uncited Supplementary Material 1. [file acmi-8-01104-s001.pdf]
